# Weather forecasts become more important for reducing mortality as the climate warms

**DOI:** 10.1073/pnas.2523372123

**Published:** 2026-04-13

**Authors:** Jeffrey G. Shrader, Stephan Thies, Laura Bakkensen, Manuel Linsenmeier, Derek Lemoine

**Affiliations:** ^a^School of International and Public Affairs, Columbia University, New York, NY 10027; ^b^Department of Economics, University of Oregon, Eugene, OR 97403; ^c^Center for Critical Computational Studies, Goethe University, Frankfurt am Main 60322, Germany; ^d^High Meadows Environmental Institute, Princeton University, Princeton, NJ 08544; ^e^Department of Economics, University of Arizona, Tucson, AZ 85721; ^f^National Bureau of Economic Research, Cambridge, MA 02138; ^g^Centre for Economic Policy Research, Paris 75007, France

**Keywords:** climate change, forecasts, mortality, early warning

## Abstract

Climate change will increase the frequency and severity of extreme heat, which is a leading cause of weather-related mortality. We combine causal estimates of how temperature forecast accuracy affects mortality in the United States with expert projections of changes in forecast accuracy over the coming century. Our analysis shows that improving short-run temperature forecasts in line with central expert projections would reduce annual heat-related mortality by about 18% by 2100, saving thousands of lives per year. If investments in earth observation networks and weather models improve forecasts in line with optimistic expert projections, annual heat-related mortality would fall by around 25% by 2100. Improving forecasts facilitates adaptation to climate change, saving more lives when climate change is more severe.

## Significance

People use weather forecasts to avoid deadly consequences of extreme weather, but the usefulness of forecasts depends on their accuracy. We find that accurate temperature forecasts already save lives, particularly during periods of extreme heat. To assess how much further these benefits might grow, we surveyed meteorologists about their expectations for forecast accuracy over the coming century. Their different expectations imply substantially different levels of protection against future heat risk. The trajectory of forecast improvement becomes increasingly consequential as climate change increases the frequency and severity of hot days: Faster improvements to accuracy could prevent thousands of deaths each year.

When people know extreme weather is coming, they can use that information to adjust their plans (1–6). Indeed, people do use weather forecasts to reduce temperature-related mortality, especially from hot weather (7). Future climate change is projected to increase mortality from hot weather and to reduce mortality from cold weather (8–12). However, these projections have not accounted for how the forecastability of weather may change over coming years and the effect this might have on weather-related harms. This omission is potentially important because forecasts have been improving steadily over recent decades (13, 14), and new investments (15, 16) and advances in machine learning (17–21) should spur further improvements.

Here, we show that improving short-run U.S. temperature forecasts can save thousands of lives per year, especially if the future climate is warmer. Improving forecasts in line with expert projections would reduce mortality on hot days by 18%. Experts believe even faster progress in forecasting is feasible, in which case heat-related mortality would fall by 25%. Making forecasts more accurate reduces mortality both with and without climate change, but because mortality is especially sensitive to forecasts on hot days, more accurate forecasts save more lives under climate change. More accurate forecasts are not a form of adaptation in themselves; instead, they facilitate day-to-day adaptation decisions by people, emergency response personnel, and other groups. Investing in weather observations, forecast model improvements, and communication of forecasts is especially valuable under more severe climate change because the adaptation that forecasts facilitate becomes more important.

**Fig. 1.**
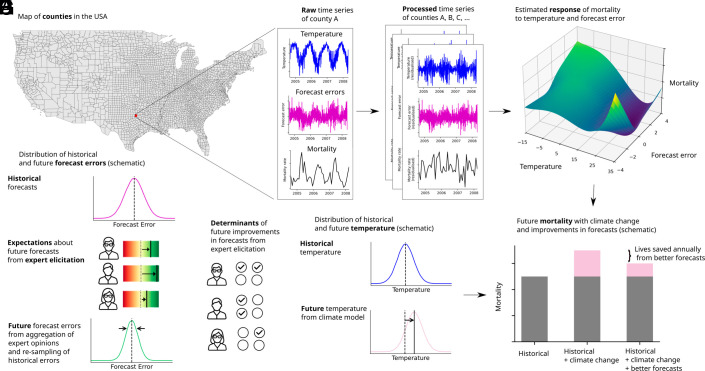
Quantification of lives saved from future forecast improvements. The figure shows the steps of the methodology. Panel (*A*) visualizes the geographical extent of the study area. Panel (*B*) illustrates the three types of data that are collected for each county in the sample for every day: temperature, forecast errors, and mortality. Panel (*C*) shows the remaining “quasi-random” variation in the time series after possible confounders are removed. Panel (*D*) illustrates the empirical model and the estimated combined effect of temperature and forecast errors on mortality. Panel (*E*) illustrates the generation of future forecast errors from an expert elicitation and a resampling of historical errors. Panel (*F*) visualizes the elicitation of contextual information about future development of forecasts from the experts. Panel (*G*) shows the distribution of future temperature, from climate models. Panel (*H*) illustrates how the empirical model and future temperature and forecast error projections are combined to quantify future lives saved from forecast improvements.

We develop a regression framework that permits the effects of realized temperatures on mortality to depend on how well temperatures were forecasted, and we combine the regression results with climate model projections of future temperatures and an new expert elicitation of future temperature forecast accuracy (Fig. 1). We study U.S. counties (Fig. 1*A*). For each county, we collect the history of realized temperature, day-ahead temperature forecasts, and mortality (Fig. 1*B*). Our regression framework accounts for the ways that mortality may differ by county and season, for trends in mortality at the county level, for statewide mortality patterns within each month of the sample, and for rainfall (*Materials and Methods*). This framework implicitly produces processed time series for each variable in each county (Fig. 1*C*). We use these processed time series to estimate the relationship between excess mortality and temperature, which we allow to vary with the errors in day-ahead temperature forecasts (Fig. 1*D*). Our key identification assumption is that the processed time series exhibit only “quasi-random” variation, so that nothing that directly affects mortality happens to covary with forecast errors. In particular, we assume that there were no unobserved dimensions of weather that both i) covaried with forecast errors but not realized temperature and ii) were lethal in their own right. Prior work has validated this assumption in U.S. mortality data by demonstrating robustness to controlling for many different weather variables (7).

We survey expert weather forecasters to find out how they think forecast accuracy will improve over this century (Fig. 1*E*) and what factors and investments could drive those improvements (Fig. 1*F*). We use their answers to construct scenarios for forecasting accuracy in 2100 (*Lower* part of Fig. 1*E*). We obtain scenarios for temperature at the end of the century from climate models’ projections (Fig. 1*G*). We use our estimated relationship between mortality, temperature, and forecast errors from Fig. 1*D* to project mortality under each climate change scenario and each forecast error scenario. Finally, we calculate the lives saved from improved forecasts under each climate change scenario as the difference between our projections of mortality with constant and improved forecast accuracy (Fig. 1*H*).

## Accurate Temperature Forecasts Reduce Mortality on Hot Days

**Fig. 2.**
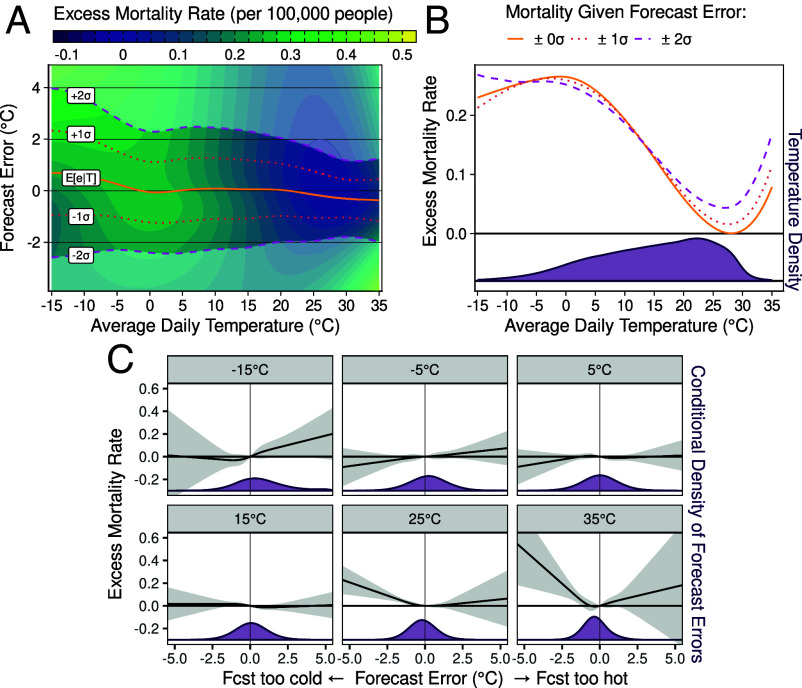
Mortality is affected by temperature and forecast errors. The figure shows the effect of realized temperature and day-ahead forecast errors on the daily excess mortality rate (per 100,000 people). Panel (*A*) shows the estimated excess mortality rate for every combination of realized temperature and forecast error. Lines colored from orange to purple indicate the average forecast error (solid orange) as well as errors 1 and 2 SDs from the mean (dotted orange and dashed purple, respectively). The shading reflects the estimated effect on mortality, from blue for a small effect to yellow for a large effect. The other panels in the figure cut through this estimated mortality response surface. Panel (*B*) plots the estimated excess mortality rate against temperature when forecast errors are 0, 1, or 2 SDs in magnitude (solid orange, dotted orange, and dashed purple respectively). The curves average over the corresponding lines in panel (*A*). The *Bottom* of the panel shows the population-weighted density of daily average temperature realizations during the sample period. Panel (*C*) shows the estimated effect of forecast errors on excess mortality at different temperatures relative to mortality at zero forecast error. The gray bars show 95% CIs. The *Bottom* of each figure shows the population-weighted density of forecast errors on days with that average temperature.

We first establish the empirical relationship between excess mortality and both forecasted and realized temperatures. Fig. 2*A* depicts the estimated surface relating these three variables. Fig. 2*B* projects the estimates onto the mortality-temperature axis for three levels of forecast error. Across our sample period, we find that both hot and cold temperatures increase mortality and that accurate forecasts reduce mortality, particularly on warm and hot days.

The combined effect of realized temperature and forecasted temperature can be decomposed into an effect of realized temperature when perfectly forecasted and an effect of forecast errors for a given realized temperature. We first consider the mortality consequences of temperature when temperature turns out to be perfectly forecasted (the solid orange lines in Fig. 2 *A* and *B*). The daily excess mortality rate due to temperature is greater than 0.2 per 100,000 people on days with an average temperature near 0 ^°^C, falling to zero at 27.8 ^°^C, then rising again to about 0.1 per 100,000 people at 35 ^°^C. The latter days are at the 99.9th percentile of days in our sample but will become more common with climate change.

Heat can be deadlier when forecasted erroneously. The dotted orange line in Fig. 2*B* shows how mortality responds to temperature when a one SD forecast error occurs, averaging over the cases when the error is too cold or too hot. The dashed purple line shows the same for a two SD error. These lines are clearly above the perfect forecast (solid orange) line on days with an average daily temperature above 20 ^°^C, indicating that forecast errors are especially deadly on the hottest days. Forecast errors also kill people on cold days with an average daily temperature below −5 ^°^C. However, because the average person in the United States experienced an average daily temperature below −5 ^°^C on only 13 d per year between 2004 and 2023 but experienced around 135 d per year with average daily temperatures above 20 ^°^C (Fig. 2 *B*, *Bottom* panel), hot days drive mortality from forecast errors even in the current climate. Prior work with individual-level data suggests that more accurate forecasts especially reduce mortality from cardiovascular disease and from accidents (7).

Comparing the dotted and dashed lines in Fig. 2*B* to the solid orange line shows the effect of forecast errors on mortality, averaging over whether the forecast turned out to be too hot or too cold. If too-moderate forecasts were harmful but too-extreme forecasts were beneficial, then mortality could have turned out to be approximately the same, on average, on days with erroneous forecasts of a given magnitude as on days with perfect forecasts. That is not what we find, indicating that the effect of forecast errors on mortality must be nonlinear. Projecting the estimated mortality-temperature-forecast relationship from Fig. 2*A* onto the mortality-forecast error axis (Fig. 2*C*) disentangles the effect of forecasts that turned out to be too cool (negative forecast errors) from those that turned out to be too warm (positive forecast errors). Too-cool forecasts do not significantly affect mortality on the coldest days, but too-warm forecasts are hazardous on the coldest days. In contrast, too-cool forecasts are especially deadly on hot days whereas too-warm forecasts do not significantly affect mortality on those days. And forecast errors of either type do not significantly impact mortality on days with more moderate temperatures. The nonlinear, convex effects of forecast errors on cold and hot days explain why forecast errors increase mortality on average in Fig. 2*B*.

Finally, we note that forecast accuracy is itself heterogeneous over the temperature distribution. On days with moderate temperatures, the average forecast error is approximately zero (solid orange line in Fig. 2*A*). At more extreme temperatures, the average forecast error becomes nonzero, with forecasts tending to be too warm (positive average error) when the weather is cold and forecasts tending to be too cool (negative average error) when the weather is hot. These average errors at extreme temperatures do not result from forecasters skewing their forecasts. The mean error is zero when we condition on the forecast rather than the realized temperature (*SI Appendix*, Fig. S1), implying that the forecast is unbiased. Instead, the average errors on days with extreme realized temperatures reflect that days with the highest and lowest realized temperatures tend to be days that overshot their forecasts ex post, as days that undershot their forecasts tend not to end up as extreme.

This pattern matters because when researchers focus on days that turned out to be extremely hot or cold ex post without controlling for forecasts, they implicitly select for days on which forecasts were too mild and implicitly select against days on which forecasts were overly extreme. As a result, estimates of mortality from extreme temperatures in previous studies that did not consider forecasts reflect the effect of extremes that tend to also be extreme relative to forecasts, which may not be representative of mortality from future days with the same temperature as the climate changes. We avoid biases from this selection by explicitly including forecasts in our empirical model.

## Experts Expect Temperature Forecasts To Become More Accurate

**Fig. 3.**
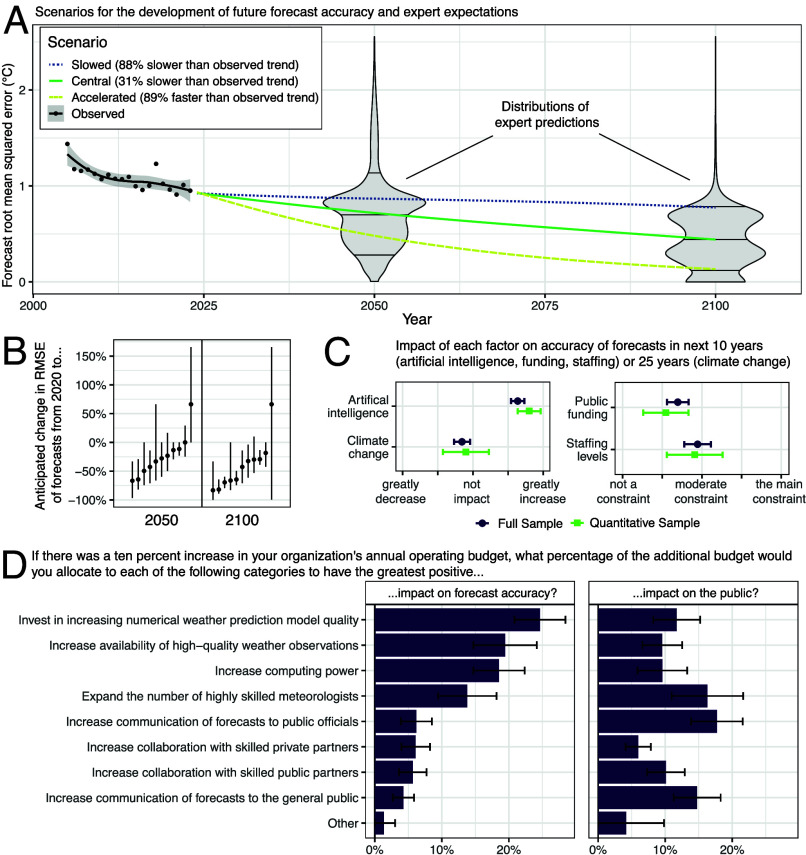
Experts expect temperature forecasts to continue improving. Panel (*A*) shows observed historical (2005–2023) and possible future scenarios for annual average forecast accuracy (measured by root mean squared error) in the United States. The violin plots for 2050 and 2100 indicate the aggregate distribution of expert expectations about the future development of forecast accuracy. Horizontal lines inside the violins indicate 10th, 50th, and 90th percentiles of the distributions. Panel (*B*) shows raw expert responses for the predicted percentage change in forecast accuracy between now and both 2050 and 2100. Panel (*C*) shows expert answers regarding factors that could have an impact on future forecast accuracy. The purple lines depict results from our full sample of 48 professional weather forecasters, and the green lines depict results from the 11 professional forecasters who provided quantitative answers that we used to construct the scenarios in panel (*A*). Panel (*D*) visualizes expert answers on how they would spend funds to improve forecast accuracy or improve the value of forecasts to the public. In the full sample, 8 out of 48 forecasters did not answer each of the questions, see *Materials and Methods* for details on sample selection.

In order to project future forecast accuracy, we surveyed expert forecasters from around the United States (*Materials and Methods*). Temperature forecasts have steadily improved in the United States over recent decades (13, 22). Measuring forecast accuracy by root mean square error (RMSE, with lower values indicating greater accuracy), we find that 1-day-ahead temperature forecast accuracy improved by 34% between 2005 and 2023 (Fig. 3*A*). Most experts expect continued improvement in forecasting accuracy from now until 2050 and again from 2050 until 2100, with the median expert projecting that forecast RMSE will fall by around half by 2100 (Fig. 3 *A* and *B*). Relatively pessimistic experts expect only small improvement, with one expert even expecting forecast accuracy to degrade substantially. The most optimistic experts expect forecast RMSE to fall by nearly four-fifths.

We use the professional forecasters’ responses to construct scenarios for how temperature forecast RMSE might evolve in the future. We construct the scenarios by calculating the change in the observed, historical trend in forecast RMSE required for RMSE to hit different percentiles of the distribution of expert predictions for 2100. To fit the historical trend, we use a linear model of percentage changes in forecast accuracy. It explains the historic data equally well as a model of absolute changes and ensures nonnegative extrapolations of forecast RMSE (*Materials and Methods*).

We distinguish three scenarios (Fig. 3*A*). The “central” scenario (green solid line in Fig. 3*A*) assumes that forecast accuracy improves over the century so that it passes through the median forecaster’s projected accuracy in 2100. Although this scenario involves a halving of RMSE relative to present-day values (Fig. 3*B*), it still represents a 31% slowdown in forecast improvement relative to the trend observed over 2005–2023 (average trend of solid black circles in Fig. 3*A*). The “slowed” scenario (blue dotted line) has forecast accuracy improve only to the experts’ 90th percentile in 2100. This scenario represents a deceleration of the observed, historical trend by 88% and leaves forecast accuracy almost unchanged relative to today. Finally, the “accelerated” scenario (yellow dashed line) has forecast accuracy improve all the way to the experts’ 10th percentile in 2100. This scenario nearly doubles the rate of forecast improvement observed over 2005–2023. The scenarios are not forced to pass through any percentiles of the 2050 expert prediction distribution. Despite this, the central scenario still passes close to the median of the 2050 predictions.

We also assess the factors that experts believe are important for future forecast accuracy (Fig. 3*C*). There has been much excitement about the prospects for machine learning and artificial intelligence to improve forecasts (17–21, 23), and there have also been questions about how climate change will affect forecast accuracy (24–27). Our experts believe that artificial intelligence will improve forecasts but do not expect climate change to greatly affect the accuracy of short-range forecasts (Fig. 3*C*). The expert forecasters also expect staffing to moderately constrain forecast accuracy over the next decade and public funding to more weakly constrain accuracy over the next decade (Fig. 3*C*). These answers were provided prior to the early 2025 cuts to the US National Weather Service staffing. In showing responses for the full sample of respondents and also for the subset who provided quantitative projections of forecast accuracy (purple circles and green squares, respectively), Fig. 3*C* allows us to test for selection among the experts who provided quantitative projections. The similarity of the answers in Fig. 3*C* visually suggests little evidence of selection. And when formally testing for selection using an omnibus F-test with randomization inference (28), we find no significant difference between experts who started versus completed the survey (*P*-value of 0.109, see *SI Appendix*, Table S1). Corroborating this evidence, the spread of responses in Fig. 3*B* suggests that experts indeed were not selecting into completing our survey on the basis of shared beliefs about future forecast accuracy.

Asking how forecasters would allocate a budget to improve forecast accuracy (Fig. 3*D*, *Left* panel), we find that the top three approaches to improving forecast accuracy all involve improving models, data, or computation, with the fourth involving staffing. In contrast, two of the top three ways of helping the public (Fig. 3*D*, *Right* panel) involve communication. Consistent with staffing being considered a constraint, experts consider increasing the number of highly skilled meteorologists to be the second-most impactful investment for the public.

We also asked two open-ended questions about what could improve or degrade forecasts by 2050. Common categories of responses are summarized in *SI Appendix*, Fig. S14. For both questions, the experts emphasize the importance of high-quality observations and input data. Experts identify reductions in data gathering as a particularly important source of potential forecast degradation: Terms related to data appear in nearly half of responses, which is the most frequent response among any of the categories we consider. The next-most frequent category of responses for both questions involves artificial intelligence and machine learning. Experts see promise for these models to improve forecasts going forward, with almost the same number of responses including discussion of data as of artificial intelligence (just over half of responses for the former and just under half for the latter). Experts caution that artificial intelligence models must be based on good data inputs if one wants accurate forecast results, and many experts emphasize that artificial intelligence models that do not have any humans in the loop could lead to worse forecasts. Overall, experts are in greater agreement about what would improve forecasts than what would degrade forecasts. The average semantic similarity (*Materials and Methods*) between responses about what would improve accuracy is 82% but only 74% for responses about what would degrade accuracy (t-statistic for the difference: 23.4).

## Climate Change Makes Improving Temperature Forecasts More Important

We now use the estimated response of mortality to temperature and forecast errors over our sample period, combined with the expert projections of future forecast accuracy and model-based projections of warming under climate change, to project the lives saved by the end of the century from improvements to day-ahead temperature forecasts. Relative to a scenario in which forecasting accuracy does not change further, we consider four scenarios for how forecast accuracy will evolve by 2100: the three scenarios for improved forecast accuracy described in the last section and a “zero error” bounding scenario in which day-ahead temperature forecasts become perfect by the year 2100. We also consider four scenarios for how temperature will evolve over the century: a no climate change scenario in which temperatures in 2095–2100 are identical to observed values in 2015–2020, the SSP2-4.5 scenario in which the contiguous United States warms by 1.6 ^°^C (relative to the observed 2015–2020 population-weighted average temperature of 15.0 ^°^C), an intermediate SSP3-7.0 scenario in which the contiguous United States warms by 2.7 ^°^C, and a hotter SSP5-8.5 scenario in which the contiguous United States warms by 3.8 ^°^C (29–31).

**Fig. 4.**
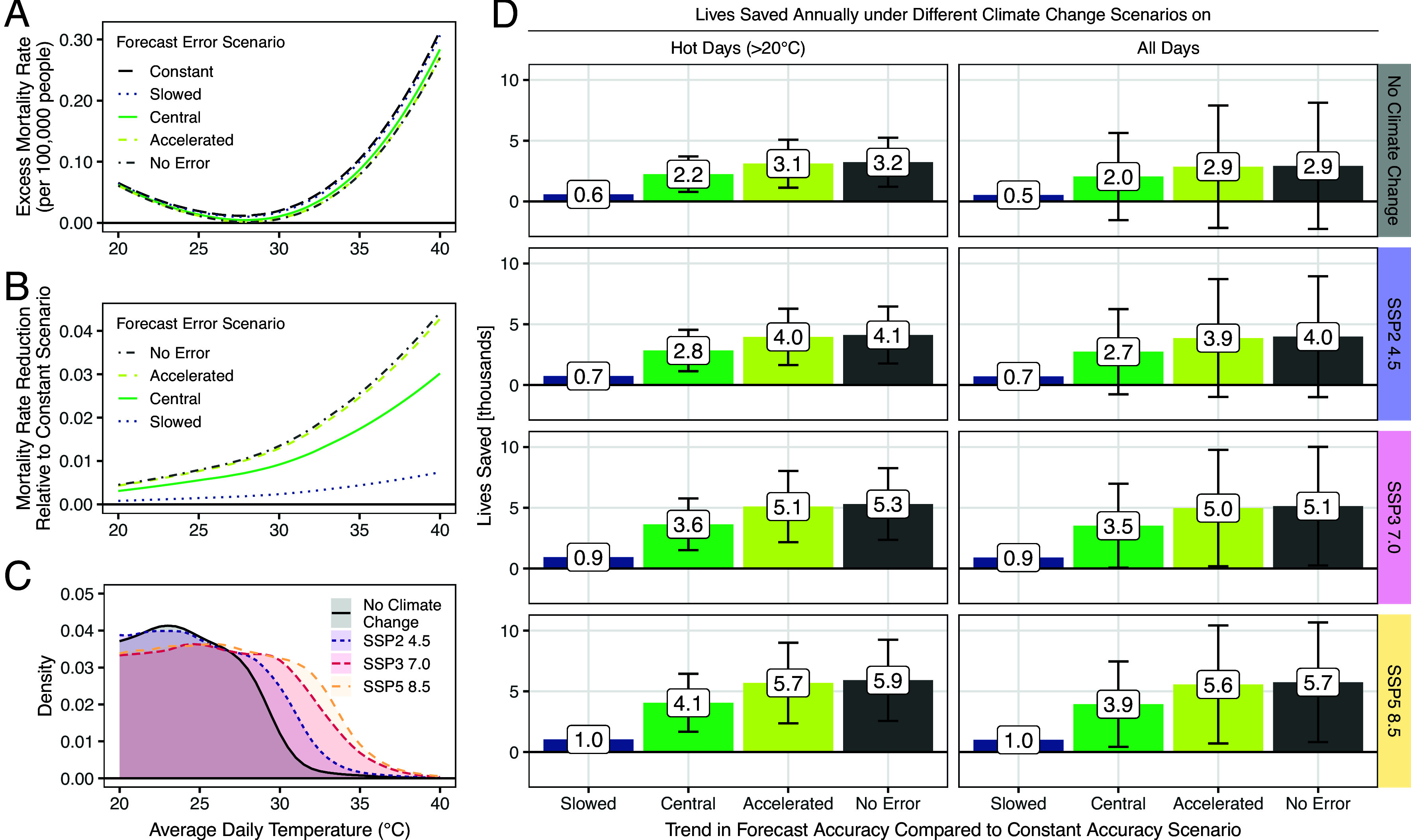
Developments in forecast accuracy become more important with climate change. Panel (*A*) shows the average daily excess mortality rate on hot days as a function of temperature for different forecast error scenarios. Panel (*B*) shows the difference between the mortality response in the constant forecast error scenario and the response in each other forecast error scenario. The difference corresponds to the reduction in heat-related mortality due to the respective improvement in forecast accuracies. Panel (*C*) shows the population-weighted distribution of average daily temperatures above 20 ^°^C under each of the year 2100 climate change scenarios. Panel (*D*) depicts annual lives saved in the contiguous United States in the year 2100 under each climate change and forecast error scenario when compared to the constant forecast error scenario. The first column in panel (*D*) shows lives saved solely on hot days exceeding 20^°^C and corresponds to integrating county-level versions of the curves in panel (*B*) over county-level versions of the temperature distributions shown in panel (*C*). The second column of panel (*D*) shows lives saved across all days in a year. The bars within each plot vary the forecasting scenario, and the error bars depict 95% CIs based on SEs from regression Eq. **1**.

We first consider how improvements in forecasting, which narrow the forecast error distribution, change the effect of realized temperature on the mortality rate (Fig. 4*A*). The curves show that for all four forecast accuracy scenarios, the excess mortality rate on hot days is higher when forecasts are less accurate. Fig. 4*B* shows how the average mortality rate at hot average daily temperatures would fall if forecasts improved. Because forecast errors are deadly in hot weather, improving forecasts reduces heat-related mortality, and because forecast errors are especially deadly in the hottest weather, mortality in the most extreme heat is especially sensitive to forecast projections.

To quantify the lives saved annually in the United States from improved forecasting (Fig. 4*D*), we integrate county-level curves analogous to those shown in Fig. 4*B* over county-level temperature scenarios analogous to those shown in Fig. 4*C*. (For exposition, Fig. 4 *B* and *C* show population-weighted averages of these curves across all counties in the contiguous United States) We thereby obtain the expected number of lives saved from forecast improvements under different climate change scenarios. We report lives saved on days with average temperature above 20 ^°^C (*Left* column in Fig. 4*D*) and lives saved on all days (*Right* column).

Without future climate change (*Top* row in Fig. 4*D*), forecast improvements save around 500 lives per year in the slowed scenario (blue bars in Fig. 4*D*) and around 2,000 lives per year in the central scenario (green bars). These savings are driven by avoided mortality on hot days, reflecting that mortality is most affected by forecast errors on days exceeding 20 ^°^C (see Fig. 2 above). Under the accelerated scenario (yellow bars), forecasting improvements would save around 2,900 lives per year, which is close to the total achievable from perfect day-ahead forecasts (gray bars). Note that all of these values are based on 2004 U.S. population (see *Materials and Methods* for a discussion of why we fix population to 2004 levels). If the U.S. population increases in line with Census or United Nations population projections by 2100, the lives saved by the central forecast projection would be 2,500 or 2,900, respectively (32–34).

We conducted a variety of robustness checks (*SI Appendix*, Fig. S15). These change the population weighting, drop any county with imputed mortality data, exclude sample years after 2019, remove the debiasing procedure for processing forecast errors, replace our weather data source with raw station data, and simultaneously estimate the effect of 1- and 3-day-ahead forecasts. In all cases, we find that forecast errors on hotter days tend to increase mortality.

Improving forecasts accuracy saves more lives in the more extreme climate change scenarios because forecasts are most beneficial on very hot days (rows two to four of Fig. 4*D*). For example, relative to no climate change, the central forecast scenario avoids 600 more deaths on hot days under SSP2-4.5, 1,400 more deaths on hot days under SSP3-7.0, and 1,900 more deaths under SSP5-8.5. Improving forecasts faster saves more lives. For example, achieving the central forecast scenario instead of the slowed scenario avoids 2,000 deaths per year in the SSP2-4.5 scenario and avoids 2,600 deaths per year in the higher-warming SSP3-7.0 scenario.

Monetized using a standard value for mortality risk reduction in U.S. federal government benefit–cost analyses (35), achieving the central forecast accuracy scenario relative to the slowed scenario under SSP3-7.0 warming is worth more than $30 billion per year by 2100 (in 2024 dollars). Our experts identify four areas they would prioritize for funding in order to increase the accuracy of future temperature forecasts: numerical weather models, near-surface weather observations, computing power, and hiring skilled meteorologists (Fig. 4*D*). Failing to invest in these areas—or introducing other policies that cause forecast improvements to fall behind the expected trend scenario—may cost tens of billions of dollars per year in increased mortality risks.

Additional results show the overall changes in mortality between 2020 and 2100 due to the combined effects of climate change and improved forecasts (*SI Appendix*, Figs. S5 and S6). Mortality from heat falls by 18% in the central scenario and by 25% in the accelerated scenario.

Forecast improvements largely offset projected increases in heat-related mortality due to climate change. For example, the 4,000 lives saved in the accelerated scenario under mild climate change (SSP2-4.5) would more than fully offset the expected increase in heat-related mortality. For the higher-warming SSP5-8.5 scenario, accelerated forecast progress reduces the expected increase in heat-related mortality by about three quarters, from 7,500 to 1,800 lives annually.

**Fig. 5.**
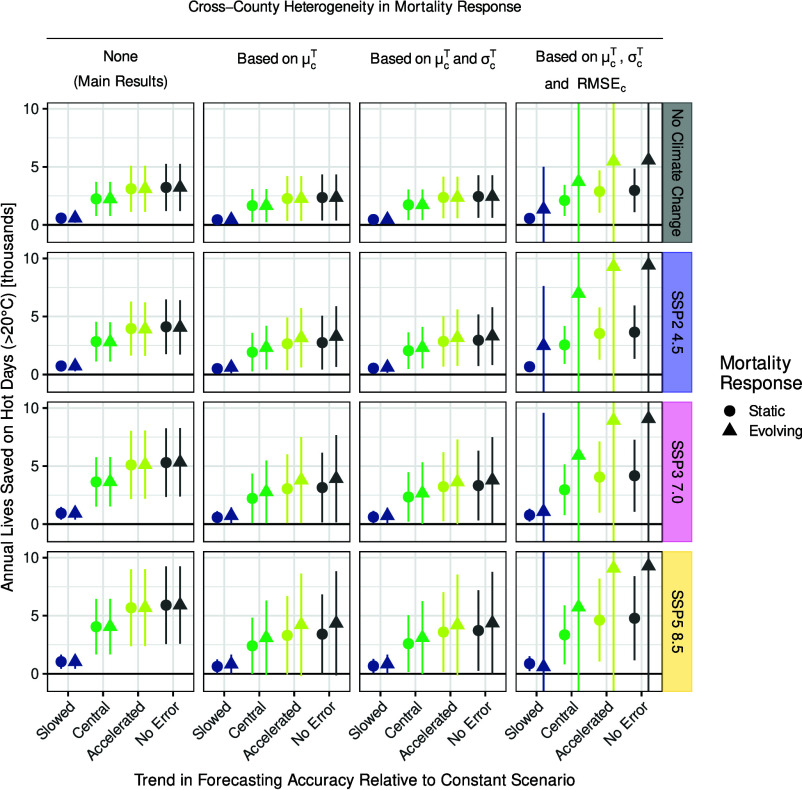
Robustness of results to heterogeneous and evolving mortality response. Column 1 replicates the main results for hot days exceeding 20 ^°^C from Fig. 4*D*. Column 2 allows the response function to vary across counties based on a county’s average temperature $\mu_{c}^{T}$. Column 3 also allows heterogeneity based on a county’s daily temperature variability $\sigma_{c}^{T}$. Column 4 also allows heterogeneity based on a county’s temperature forecast accuracy $RMSE_{c}$. In all specifications, circles indicate results for mortality response functions that are static over time. Triangles show results for mortality response functions that can evolve by the end of the century with changes in the respective county characteristics.

We have allowed the effects of forecast errors on mortality to vary with realized temperature, so that the effect of forecast errors on mortality can differ on hot days versus mild or cold days. Fig. 5 assesses the robustness of our projected effects of forecast scenarios on hot-day mortality to allowing more general forms of state dependence (see *Materials and Methods*, and see *SI Appendix*, Fig. S10 for total mortality). The leftmost column reproduces results from the *Left* column of Fig. 4*D*, with rows varying the climate change scenario and horizontal categories within a column varying the forecast accuracy scenario. The second column allows the effects of realized temperatures and forecast errors to vary by counties’ average temperatures. People in hotter locations may be better adapted to heat and may have different uses for forecasts of heat. The third column allows the effects of realized temperature and forecast errors on mortality to also depend on the daily variability of temperature within a county over time, measured as the root mean squared difference between today’s and yesterday’s temperature in the county. This dependence captures how agents may gain more information from forecasts when temperature is variable or may invest in resilience to a range of temperatures when temperature is variable. The circles hold each county’s estimated relationship constant into the future, and the triangles allow the temperature-mortality relationship to evolve as climate change alters each county’s average temperature (10, 11) and temperature variability. In either case, the lives saved from better forecasts are not substantially different from our base specification, suggesting that our main results are robust to permitting the mortality-temperature-forecast function to evolve in response to future changes in the local climate.

The rightmost column permits the effects of realized temperature and forecast errors on mortality to also depend on the average accuracy of a county’s forecasts over time, measured by RMSE. When conditioning on daily temperature variability, this dependence captures how agents may pay more attention to forecasts and may judge a given level of error as more unexpected when forecasts tend to be of higher skill. When agents’ responses are static (circles), results are again substantially unchanged. But results become noisy when agents’ responses evolve with RMSE over time (triangles), so that agents use forecasts differently when they are aware of forecasts having improved. The reason is that RMSE falls so much over the century that our projections take agents’ responses far beyond the range of the data (*SI Appendix*, Fig. S7). Reassuringly, however, the point estimates are qualitatively consistent with our main results.

We have found that mortality on hot days is especially sensitive to forecast errors. Moving beyond reduced-form relationships with average temperature or temperature variability, we now investigate how our results are specifically affected by air conditioning adoption. To the extent that this adoption is driven by counties becoming warmer over time, it may be captured by our analysis of how the response function evolves as average temperature changes (Fig. 5). However, adoption of air conditioning could also be driven by changes in income, technology, or the building stock that are uncorrelated with average temperature, temperature variability, or forecast accuracy. *SI Appendix*, Fig. S8 plots the county-level residualized excess mortality rate from forecast errors against residualized air conditioning take-up (*Materials and Methods*). It shows that mortality from forecast errors is, if anything, increasing in the unexplained portion of air conditioning take-up. An increasing relationship indicates that adaptation enabled by more accurate day-ahead forecasts is complementary to air conditioning. Such a result is plausible: Time use is an important channel by which people respond to hot weather and to forecasts of hot weather (7, 36), and people may be better able to adjust their schedules to reduce heat-related mortality when they have a cool house to stay in. Because air conditioning adoption is likely to increase over time, these results suggest that any future air conditioning take-up beyond what we account for in Fig. 5 would, if anything, further increase the lives saved from more accurate forecasts.

## Discussion

People use weather forecasts to mitigate health hazards from hot weather. By increasing the frequency of the hottest days, climate change will make accurate forecasts more beneficial and inaccurate forecasts more harmful. Improving forecast accuracy is therefore especially important under climate change. Past improvements in forecast accuracy have come via public funding for Earth observations, data sharing networks, modeling advances, and computational resources (13, 22). Further funding could be critical to limiting mortality from extreme temperatures, helping to achieve one of the objectives in the National Weather Service’s mission (37).

Our results have implications for projecting future damages from climate change. Current estimates of the social cost of carbon—the present discounted value of damages from emitting carbon dioxide at a given point in time—are heavily influenced by heat-related mortality (35). Those estimates do not take potential changes in weather forecast accuracy into account. We show that it is important to distinguish between expected heat and unforecasted heat when projecting mortality, and we show that maintaining the historical rate of progress in weather forecasting could reduce the additional mortality from hot days in the United States.

Several factors could render our projections inaccurate. First, we project forecast errors under different climate change scenarios by combining a statistical model relating historical forecast errors to temperature and location with expert projections of future forecast accuracy. The statistical model passes robustness checks (*SI Appendix*, Fig. S15*B*), and our experts’ nearer-term projections are consistent with recent advances in modeling. Progress beyond that captured in our “accelerated” scenario would not strongly affect our conclusions, as we do not find many more lives saved in a (physically impossible) scenario with perfect forecasts. The greater risk is that forecast accuracy degrades in the future. As the experts point out, weather forecast accuracy depends on the quality and coverage of input data. Reductions in weather and climate data or restrictions on data sharing could increase lives lost from forecast errors.

Second, people and agencies may change how they consume or use forecasts. In recent years, widespread diffusion of smartphones has expanded access to quality forecasts. By increasing use of forecasts, these types of technologies may increase the benefits of accurate forecasts and amplify the hazards from inaccurate forecasts. We do not find evidence of a trend in the effect of forecasts on mortality within our sample (*SI Appendix*, Fig. S16). Nonetheless, future work should consider how new communication technologies and interfaces affect the benefits of improving forecast accuracy. In addition, the US National Weather Service increasingly views its role as going beyond the production of forecasts to include reaching out to private actors and public agencies to help them make decisions based on forecasts (38). Changes in this type of coordination could amplify or undercut the benefits of more accurate forecasts.

We have focused on the benefits of forecasts for reducing temperature-related mortality under climate change. Prior work shows that U.S. citizens are willing to pay billions of dollars per year for the mortality reduction that comes from improved forecasts, even in the current climate (7). Recent work has also quantified the economic benefits of forecasts for construction (39), transportation (4), and hurricane preparedness (5), among other applications (40). How these other uses add to forecasts’ value in facilitating adaptation depends on the extent to which hotter weather drives forecasts’ value in these applications.

Over recent decades, forecasts have improved at similar rates between 1-d and 10-d horizons (14). Just as day-ahead and multiday forecasts improved jointly over past decades, the types of investments in models, infrastructure, and data that would improve future day-ahead forecasts are also likely to improve future multiday forecasts. Our results capture the value of such investments, as we find that our estimates capture the combined effects of both day-ahead and multiday forecasts (*SI Appendix*, Fig. S15). Future work could do more to consider whether investments can be targeted to improving one forecast horizon rather than another and, if so, to disentangle effects at different forecast horizons and consider the allocation of research support across horizons.

Governments have recently prioritized improving seasonal-scale forecasts (15, 16). Forecast users throughout the economy do pay attention to forecasts at the seasonal horizon (3, 41). The effect of these longer-run forecasts on mortality is not well known, nor is the interaction between short-run, multiday forecasts and longer-run, seasonal forecasts. Short-horizon forecasts will be especially valuable if people have ready access to actions with low dynamic adjustment costs, and it may be that forecasts at different horizons substitute for each other if better short-run forecasts enable users to correct course without long-run investments (42). This would be the case, for example, if the ability to reschedule work reduces the risk of taking an outdoor job. But it may also be that forecast users make long-run investments based on long-run forecasts only when they know that they can make such course corrections. For example, air conditioning could become more valuable if people expect to be able to reschedule work hours to avoid outdoor heat. As multiweek and seasonal forecasts mature and diffuse, future estimates of the benefits of forecasts for mortality at different forecast horizons could help guide policy investments.

## Materials and Methods

### U.S. Weather and Weather Forecasts.

Our two primary explanatory variables are daily average temperature and forecasted temperature. Temperature forecasts come from the National Weather Service (NWS) National Digital Forecast Database (NDFD). This database contains the forecasts that users see on the NWS website (weather.gov). These forecasts result from integrating numerical weather predictions, other computational processing, and expert judgment from NWS meteorologists. We focus on the one-day-ahead forecast and on daily minimum and maximum temperature point forecasts, from which we calculate daily average temperature by taking the average of the two measures. We use the noon UTC forecast run. We use forecast data from June 6, 2004, onward, which is the universe of data available in the NDFD containing both minimum and maximum temperature forecasts. Roughly 5% of the county-day values have missing values in the database. We interpolate these observations using the nearest available forecast. The NDFD stores the forecasts on a consistent spatial grid with resolution of 2.5 km or 5 km, depending on the time period. We aggregate the forecasts to the county level by taking the population-weighted average, based on the 2010 population grids from ref. 43.

For weather realizations, we use PRISM (Parameter-elevation Regressions on Independent Slopes) Climate Group data (44). PRISM combines weather station observations with a regression-based interpolation procedure that accounts for weather gradients such as elevation, weather inversions, rain shadows, and coastal proximity. The output is daily measures of weather on a consistent 4 km resolution grid across the country. We aggregate the gridded measures to the county level using the same procedure as the forecasts. We calculate a day’s average realized temperature by averaging the day’s minimum and maximum temperature. For robustness, we also gathered weather data from GHCN weather stations and aggregated it to daily, county-level measures following ref. 45.

### Mortality.

The primary outcome we study is mortality. Mortality data come from the CDC’s WONDER Online Database (https://wonder.cdc.gov/). It contains records of all vital events that occurred in the United States from 2004 to 2022, recorded at the county-month level. Data for counties with a small number of vital events are suppressed by the CDC due to privacy concerns. See *SI Appendix*, Fig. S3 for a map of suppressed counties. For counties with missing mortality rates we impute values using the monthly state-wide mortality rate. Estimation results do not change notably when dropping suppressed values from the estimation. We have also estimated the relationship between mortality, forecasts, and temperature at the monthly level using complete counts of vital events from the CDC’s restricted-access database (results available on request). The results are comparable when using these data.

From the set of all mortality events, we calculate county mortality rates per 100,000 people by dividing the total mortality each day by the county population in that year. Population figures are from the NIH Surveillance, Epidemiology, and End Results (SEER) Program.

### Air Conditioning.

Estimates of air conditioning take-up at the county-level are taken from ref. 7. In a Census Research Data Center, they use a model selection procedure and individual-level restricted access data to estimate the determinants of air conditioning take-up based on demographic and household characteristics in the American Housing Survey coupled with county-by-year climatic characteristics from ref. 46. Estimated coefficients were then applied to related county-level characteristics from the American Community Survey to estimate take-up rates. While the full dataset is a county-by-year panel from 2005 to 2017, we here use the county-level estimates in 2017 to examine the highest levels of take-up.

### Climate Projections.

Projections of future temperature come from the Climate Model Intercomparison Project, version 6 (CMIP6) Global Fluid Dynamics Laboratory (GFDL) model (47). We consider gridded daily temperature projections within the contiguous United States from 2015 through 2100 and use SSP2-4.5, SSP3-7.0, and SSP5-8.5. We aggregate the data to the county level using 2010 population grids from ref. 43.

To bias-adjust temperature projections for each scenario, we estimate a county-specific quadratic trend in average annual temperatures using observed PRISM data from 2005 to 2023 and the respective CMIP6 projection data from 2015 to 2030. For each county, we then predict the 2015 average temperature using both trends. The difference between the CMIP6-based and PRISM-based 2015 predictions serves as the bias adjustment, which we subtract from all CMIP6 projections to correct for systematic differences between observed temperatures and the respective climate scenario.

### Expert Survey.

To project future forecast accuracy and assess factors affecting that accuracy, we conducted an expert elicitation survey of professional meteorologists in the United States from January to February 2025. Previous literature has found weather forecasters to be well-calibrated to make these expert judgments, given that they make regular predictions aided by computer models and regularly receive feedback on their performance (48). These responses were part of a larger global survey of expert forecasters. We defined an expert as someone who is at least 18 y of age, works either at an official national or international meteorological organization or conducts meteorological research at a university or other research organization, and part of whose job responsibilities include an aspect of weather forecasting or numerical weather prediction. The survey was approved by the Institutional Review Board of the University of Arizona. The survey was distributed via email on behalf of the authors by respondents’ employers. Respondents were not compensated for their participation. Respondents were free to skip any questions they did not wish to answer and could exit the survey at any time.

After consenting and confirming their status as an expert, respondents were first asked a series of questions regarding drivers of future forecast accuracy including factors such as artificial intelligence, climate change, funding, and staffing levels. Respondents were also asked how they would allocate a hypothetical 10% organizational budget increase to best improve forecast accuracy and, in a separate question, to best benefit the public. They answered two open-ended questions about what might cause forecasts to get better or worse by 2050.

Next, respondents were asked quantitative questions about their views on future forecast accuracy. They were first asked to define their forecast location and preferred accuracy metric (Mean Squared Error, Mean Absolute Error, or Root Mean Square Error) and then were asked, “What was the [preferred accuracy metric] of three-day-ahead 2m temperature forecasts that your organization produced for [their forecast location] in the years 2005 and 2020?” They were then asked for their expert judgment on future forecast accuracy values for the years 2050 and 2100 for the following three scenarios: 1) worst-case, meaning a less than 1% chance that the forecast accuracy will be worse than their stated answer, 2) median case, meaning that there is a 50% chance that the forecast accuracy will be better/worse than their stated answer, and 3) best-case, meaning a less than a 1% chance that the forecast accuracy will be better than their stated answer. We asked about three-day-ahead forecasts to balance between the horizon focused on by different forecasting organizations. To match with our empirical results from the econometric model (described below), we apply these responses to changes in one-day-ahead forecasts. This is done under the assumption that the percent change in RMSE will evolve similarly for short-range forecasts. (Over our sample period, three-day-ahead forecast RMSE fell by 35.5% and one-day-ahead RMSE fell by 29.9%.) Respondents were also asked their level of confidence in their answers to reduce the chance of overconfidence (48).

A total of 48 U.S.-based meteorologists responded to at least one of the survey questions evaluated here. Of these, 40 provided valid answers to all nonquantitative questions, and 11 completed the full set of quantitative items. Fig. 3 *A* and *B* draw on all 11 quantitative responses. For Fig. 3*C*, the full-sample sizes are 44, 45, 46, and 46 for the questions on artificial intelligence, climate change, public funding, and staffing levels, respectively; the corresponding quantitative-sample sizes are 10, 10, 11, and 11. Fig. 3*D* is based on 42 valid responses, and *SI Appendix*, Fig. S14 *A* and *B* use 47 and 46 responses, respectively. Based on their stated forecasting location, respondents were distributed across the United States.

The complete survey instrument can be found at https://jeffreyshrader.com/papers/Expert_elicitation_survey_final_120224.pdf. The survey was preregistered with the Open Science Foundation.

#### Econometric model.

The econometric model relates mortality to temperature and forecast errors while controlling for potentially correlated spatial or temporal confounders. Overall, the model allows us to estimate the causal effect of both realized temperature and forecasted temperature on excess mortality within a county-month.

We model the relationship between mortality, temperature, and forecast errors by relating a flexible function of the latter two variables, $f_{c}\left(T_{\mathrm{cd}},e_{\mathrm{cd}}\right)$, to county-specific mortality, where $T_{\mathrm{cd}}$ is realized temperature and $e_{\mathrm{cd}}$ is realized one-day-ahead forecast error (defined as the forecasted temperature minus the temperature realization) in county c on day d. We demean forecast errors $e_{\mathrm{cd}}$ before they enter the estimation by subtracting county-specific means. This adjusts for possible measurement error or misalignment between the PRISM realized temperature and the NDFD forecast data. Results are robust to using alternative debiasing techniques such as using means at the county × month or county × month × temperature bin levels and to using raw forecast errors.

For the temperature dimension of $f_{c}\left(T_{\mathrm{cd}},e_{\mathrm{cd}}\right)$, we use a fourth-order polynomial [matching recent work estimating the relationship between mortality and temperature (11)]. The demeaned forecast errors enter $f_{c}\left(T_{\mathrm{cd}},e_{\mathrm{cd}}\right)$ according to a natural cubic spline with internal knots at the 35th and 65th percentiles of the population-weighted day-ahead forecast error distribution, which correspond to error values of −0.4^°^C and 0.3^°^C respectively. The boundary knots are at the 5th and 95th percentiles, which correspond to error values of −1.7 ^°^C and 1.8 ^°^C. Using natural cubic splines allows the impacts of forecast errors on mortality to be nonlinear in the support of the data, with the impacts of forecast errors beyond the boundary knots approximated by a linear function. The effects of forecast errors may also vary with realized temperature, both because adaptation will be different at different temperatures and because agents’ beliefs about forecast accuracy and reliance on forecasts may differ by temperature. To capture this potential state dependence of the relationship between mortality and forecast errors, we incorporate a quadratic interaction term between the natural cubic spline of forecast errors and temperatures in $f_{c}\left(T_{\mathrm{cd}},e_{\mathrm{cd}}\right)$. Overall, the procedure results in a 13-dimensional basis vector $X_{\mathrm{cd}}$, with the first four entries containing the fourth-order polynomial in $T_{\mathrm{cd}}$ and the remaining nine entries containing the natural cubic spline basis of $e_{\mathrm{cd}}$ and its interactions with $T_{\mathrm{cd}}$ and $T_{\mathrm{cd}}^{2}$. After the spline transformation of temperatures and forecast errors, the model is linear in parameters and can be expressed as $f_{c}\left(T_{\mathrm{cd}},e_{\mathrm{cd}}\right)=X_{\mathrm{cd}}^{\prime }\beta$.

To match the daily temperature and forecast data with the monthly mortality observations, we aggregate the $X_{\mathrm{cd}}$ vector to the monthly level by summing over all days within year-month t: $X_{\mathrm{ct}}=\sum_{d\in t}X_{\mathrm{cd}}$. This is a standard procedure for estimating the effect of weather observed at high frequency on outcomes that are observed at lower frequency (11, 46). When a temporal separability assumption holds, estimating the monthly model based on summing over daily values returns the same estimates as the daily model we could estimate if we observed outcomes on a daily level. This specification accounts for harvesting within each calendar month but does not account for mortality displacement across months. This means that we allow, on average, for just over two weeks of harvesting following a temperature shock or forecast error. Previous research shows that temperature impacts largely fall to zero within a few days for hot weather and two weeks for cold weather (49) and that effects of forecast errors stabilize after a few days (7).

The estimating equation is

[1] $$\begin{array}{c} m_{\mathrm{ct}}=X_{\mathrm{ct}}^{\prime }\beta +g\left(P_{\mathrm{ct}}\right)+\lambda_{\mathrm{cm}}+\alpha_{\mathrm{cm}}\times t+\rho_{\mathrm{st}}+\varepsilon_{\mathrm{ct}}, \end{array}$$

where $m_{\mathrm{ct}}$ is county c’s monthly mortality rate in year-month t. We isolate excess mortality by controlling for location and time fixed effects. The $\lambda_{\mathrm{cm}}$ are county-month fixed effects. For each month m, these absorb all location-specific factors that are fixed over the sample period, including geography, infrastructure, and governance. These also absorb all location-specific and persistent seasonal effects. The county-month trend $\alpha_{\mathrm{cm}}$ absorbs any consistent evolution in a county’s mortality rate within a season. Year-month-state fixed effects $\rho_{\mathrm{st}}$ partial out all confounders common across counties within a year-month and state combination, including nonlinear and time-varying economic or health patterns that arise at the state-level. We also control for measures of above- or below-median rainfall ($g(P_{\mathrm{ct}})$). Altogether, we estimate mortality in excess of what would be expected based on county, state, season, time period, and rainfall.

The $\varepsilon_{\mathrm{ct}}$ is an error term. We cluster all SEs at the weather forecast office (WFO) level in order to account for unobserved correlation within a WFO, both over time and across its counties. There are 116 WFOs in our study region.

We weight the regression using 2004 (beginning of sample) county population. We fix the weights to the 2004 level to prevent endogenous migration from affecting the weights.

The main coefficient vector of interest is β, a 13×1 dimensional vector corresponding to the elements of the temperature-forecast error basis, which parameterizes the mortality-temperature-forecast-error response surface.

As noted above, our main regression framework accounts for county- and season-specific mortality effects, county-level trends in mortality, and state-by-month mortality patterns, in addition to rainfall. Our key identification assumption is that, after conditioning on observables and our rich set of fixed effects, forecast errors exhibit only “quasi-random” variation, so that nothing that directly affects mortality happens to covary with forecast errors. In particular, we assume that there were no unobserved dimensions of weather that both i) covaried with forecast errors but not realized temperature and ii) were lethal in their own right. Prior work has validated this assumption in U.S. mortality data by demonstrating robustness to controlling for many different weather variables (7).

In robustness checks, we incorporate additional state dependence of the mortality response function by allowing it to vary across counties. To that end, we estimate a county-specific coefficient vector $\beta_{c}$ which can vary according to the annual average temperature ($\mu_{c}^{T}$), the average daily temperature variability ($\sigma_{c}^{T}$), and the average accuracy of one-day-ahead temperature forecasts ($RMSE_{c}$) in county c:

[2] $$\begin{array}{c} \beta_{c}=\overset{¯}{\beta }+\mu_{c}^{T}\cdot \gamma_{T}+\sigma_{c}^{T}\cdot \gamma_{\sigma }+RMSE_{c}\cdot \gamma_{\mathrm{RMSE}}. \end{array}$$

Here, each γ vector of coefficients is of dimension 13×1, so each component of the estimated mortality response surface is allowed to vary linearly with the respective county characteristic. To prevent counties with outlier characteristics from having a disproportionate impact on estimation results, we Winsorize $\mu_{c}^{T},\;\sigma_{c}^{T},\;\text{and}RMSE_{c}$ at their respective population-weighted 5th and 95th percentiles. We define daily temperature variability as the root mean squared error of a naive 1-day-ahead temperature forecast using the previous day’s temperature, i.e. $\sigma_{c}^{T}=\sqrt{\frac{1}{n}\sum_{d}{\left(T_{\mathrm{cd}}-T_{c,d-1}\right)}^{2}}$. And we define $RMSE_{c}$ as $\sqrt{\frac{1}{n}\sum_{d}{\left(T_{\mathrm{cd}}-f_{\mathrm{cd}}\right)}^{2}}$.

In the robustness checks, we add each dimension of heterogeneity consecutively. We first add the interaction with average county temperature; we then add daily temperature variability; and we finally allow for heterogeneity along all three dimensions of county characteristics. Simultaneously allowing for heterogeneity to daily temperature variability and forecast accuracy means that the mortality response function depends on the skill of temperature forecasts in county c.

In contrast to the average mortality-temperature-forecast-error response surface estimated in Eq. **1**, estimates of cross-county heterogeneity using Eq. **2** should not be interpreted as causal (50–52). Causal identification would require quasi-random variation in county average temperatures, daily temperature variability, and average forecast accuracy. However, such variation is difficult to find. We therefore follow related literature (10, 11) in treating our estimates of cross-county heterogeneity as suggestive of the effects of changing variables via climate change or improved forecasting technology.

To examine the association between excess mortality rate from forecast errors and rates of air conditioning take-up not captured by the heterogeneity modeled in Eq. **2**, we first residualize both variables by regressing them each on county-specific average temperature ($\mu_{c}^{T}$), temperature variability ($\sigma_{c}^{T}$), and forecast accuracy ($RMSE_{c}$). To minimize the effect of outliers, we include counties with residualized air conditioning take-up rates between the 2.5th and 97.5th percentiles. We also omit the four lowest residualized excess mortality counties as they are low-population, cold outliers. We focus on excess mortality from forecast errors for hot days (>20 ^°^C) weighted by number of days per year in each 1 ^°^C bin above 20 ^°^C. We plot the cross-sectional relationship and the locally weighted scatterplot smoothing (lowess) relationship. As above, this relationship depicts a cross-sectional association that may not be causal.

#### Projecting future forecast errors.

To project future mortality as a function of temperature and forecast errors, we need to simulate future day-ahead forecast errors given climate projections of daily average temperatures. Analyzing past forecast errors in the period from 2005 to 2023 reveals that forecast errors are not normally distributed conditional on temperature; tend to be positively skewed on days that turn out to be cold and negatively skewed on days that turn out to be hot; and exhibit substantial excess kurtosis at high temperatures (meaning that although typical forecast errors are lower on hot days, the error distribution has thicker tails on those days). Conditional forecast error distributions also have nonzero means at extreme temperatures and SDs that depend on temperature and location (*SI Appendix*, Fig. S4). We further observe a strong decreasing annual trend in the SD of forecast errors across all ranges of the temperature distribution, indicating that forecasts are getting more accurate across the temperature distribution over the sample period. When resampling future forecast errors, we aim to preserve all of these moments but allow the SD of forecast errors to follow different trends throughout the century consistent with expert expectations elicited in our survey of weather forecasters (e.g., the slowed projections leave forecast error SD almost unchanged whereas the accelerated projections reduce error SD by about 1^°^ by the end of the century).

Using daily, county-level data we implement the following procedure to project future distributions of forecast errors based on changes in temperature due to climate change as well as the expert predictions of how forecast error variance will evolve.

1. *Estimate in-sample average forecast error*: For each county c and day d, we estimate average forecast error by fitting a linear regression (weighted by county population to arrive at representative estimates) that conditions on daily average temperature and the WFO that the county is in. This gives us predicted average error for the county day, $\mu_{\mathrm{cd}}^{e}$, where
  [3] $$\begin{array}{c} \mu_{\mathrm{cd}}^{e}E\left[e_{\mathrm{cd}}|T_{\mathrm{cd}},\text{WFO}\right]=h_{\mu }\left(T_{\mathrm{cd}}\right)+\lambda_{\text{WFO}}. \end{array}$$
  Here, $h_{\mu }$ denotes a natural cubic spline of temperature with boundary knots at −15 ^°^C and 35 ^°^C and internal knots evenly spaced in 5 ^°^C steps. $\lambda_{\text{WFO}}$ denotes a WFO fixed effect. Counties tend to be smaller than WFOs.
2. *Estimate in-sample forecast error variance*: We estimate the variance of forecast errors in county c on day d conditional on temperature, WFO, and year in the observed 2005–2023 sample using the estimated means and a Gamma generalized linear model with a log link function and weights based on 2004 population:
  [4] $$\begin{array}{cc} \sigma_{\mathrm{cd}}^{2e} & E[{\left(e_{\mathrm{cd}}-\mu_{\mathrm{cd}}\right)}^{2}|T_{\mathrm{cd}},\text{WFO},\text{year}]\\ = & \exp\left(h_{\sigma }\left(T_{\mathrm{cd}}\right)+\lambda_{\text{WFO}}+\delta \cdot \text{year}\right). \end{array}$$
  The term $e_{\mathrm{cd}}-\mu_{\mathrm{cd}}$ is the difference between observed forecast errors and the average forecast errors (based on step 1). Like in step 1, $h_{\sigma }$ denotes a natural cubic spline of temperature with the same knots as $h_{\mu }$, $\lambda_{\text{WFO}}$ denotes WFO fixed effects, and δ denotes the annual trend in forecast error variance. We use a Gamma generalized linear model with log link to prevent future predicted forecast variances from becoming negative. Using a simple linear model as opposed to a generalized linear model with log link leads to almost identical in-sample fit but can produce negative predicted forecast variances for some WFOs at high temperatures. This problem becomes especially relevant when extrapolating forecast accuracies into the future. The log link also allows us to capture heterogeneity in the improvements in forecast accuracy by temperature. In absolute terms, RMSE has reduced more on cold days than on hot days throughout the sample period. However, improvements in percentage terms were similar across temperatures which is parsimoniously captured by the logarithmic specification.
3. *Project forecast errors under climate change*: Next, we use temperature projections from climate change scenarios to project how forecast errors will evolve as the climate changes. We compute standardized forecast errors ${\tilde{e}}_{\mathrm{cd}}$ from observed forecast errors in the 2005–2023 sample and the mean and variance terms estimated in the prior two steps: ${\tilde{e}}_{\mathrm{cd}}\frac{e_{\mathrm{cd}}-{\hat{\mu }}_{\mathrm{cd}}}{{\hat{\sigma }}_{\mathrm{cd}}}.$ The standardized errors have mean zero and unit variance and have the same higher-order moments (e.g., skewness and kurtosis) as the observed errors. These higher-order moments can still depend on temperature so in the projections we create a future, standardized forecast error, ${\tilde{e}}_{\mathrm{cd}}^{\left(b\right)}$, by first determining the 5 ^°^C temperature bin in which the projected future temperature under climate change falls then sampling from all historical standardized errors ${\tilde{e}}_{\mathrm{cd}}$ that were observed on a day with a temperature in the same bin. Given resampled standardized errors, we compute an imputed forecast error in county c on day d in a future year as
  [5] $$\begin{array}{c} e_{\mathrm{cd}}^{\left(b\right)}={\hat{\mu }}_{\mathrm{cd}}+{\hat{\sigma }}_{\mathrm{cd}}\cdot {\tilde{e}}_{\mathrm{cd}}^{\left(b\right)}. \end{array}$$
4. *Project changes in forecast error variance based on expert elicitation*: To model different scenarios of how forecast accuracy might evolve in the future, we vary ${\hat{\sigma }}_{\mathrm{cd}}$ by adjusting the estimated time trend $\hat{\delta }$ across scenarios based on the expert elicitation. We take the trends from the elicitation of experts’ beliefs about how forecast error variance will evolve by the end of this century, as shown in Fig. 3. Details on how these beliefs were elicited are discussed in *Expert Survey*. For our projections, we alter the trend in forecast error variance estimated in step 2 so that under SSP3-7.0, our projections align with the 10th, 50th, and 90th percentiles of the elicited forecast error distributions in 2100. The details on how we arrived at these percentiles is discussed in *Deriving Distributions of Future Forecast Errors*. We label these scenarios “slowed,” “central,” and “accelerated.”

When assessing the resampling procedure on the observed past temperature data from 2015–2020, it performs well in matching the SD of forecast errors conditional on temperature. It also matches other higher-order moments moderately well (*SI Appendix*, Fig. S4). The main exception is that our resampled errors have too little excess kurtosis—especially on hot days. Considering that forecast errors are especially deadly on hot days, this suggests that our extrapolation results will be conservative as they underestimate the fat tails of forecast error distributions on increasingly common days as the climate changes.

#### Projecting future changes in mortality.

Given the estimated econometric model, climate projections, and projected forecast errors, we project changes in mortality under different climate change and forecast accuracy scenarios. We start by estimating the excess mortality rate (deaths per 100,000 people) for each county c and day d:

[6] $$\begin{array}{c} {\hat{\mathrm{em}}}_{\mathrm{cd}}={\hat{f}}_{c}\left(T_{\mathrm{cd}},e_{\mathrm{cd}}\right)-{\hat{f}}_{c}\left({\text{MMT}}_{c},0\right). \end{array}$$

The function $\hat{f}$ is the fitted surface estimated using the econometric model described in *Econometric Model*. We evaluate this function at daily temperatures, $T_{\mathrm{cd}}$, and forecast errors, $e_{\mathrm{cd}}$, as well as at the minimum mortality temperature (MMT, the temperature between 10 ^°^C and 35 ^°^C that minimizes mortality when no forecast error is present) and zero forecast error. The difference between these two estimates yields the excess mortality on a day with suboptimal temperatures or forecast errors.

Our main model uses a response function $\hat{f}$ that does not vary across counties and thus produces a homogeneous MMT of 27.8 ^°^C. In robustness checks we allow for additional county-specific state dependence of the mortality response function, so the MMT can vary across counties based on average county temperature, daily temperature variability, and forecast accuracy. For a small fraction of counties, the additional heterogeneity results in no minimum being achieved between 10 ^°^C and 35 ^°^C. In these robustness checks, we restrict MMTs to the 90th percentile of MMTs we observe across counties and state-dependent specifications, which is at 29.5 ^°^C.

Given future temperatures $T_{\mathrm{cd}}$ and the respective imputed forecast errors $e_{\mathrm{cd}}^{\left(b\right)}$, we predict daily excess mortality rates ${\hat{\mathrm{em}}}_{\mathrm{cd}}^{\left(b\right)}$ for each climate change scenario and forecast accuracy scenario. To obtain average annual values of excess mortality, we multiply the daily rates by 2004 county population estimates, aggregate numbers up to the annual level, and then average them over six-year periods to prevent idiosyncratic annual variation from driving results. We use 2004 population in our projections to be consistent with the population weights used in the econometric model. Projections of population are available in the SSPs, but these projections do not incorporate the influence of changes in forecasts which could affect where people move and how many people end up living in different portions of the United States. Results for the no-climate-change scenario rely on averages across the annual excess mortality computed based on observed temperatures in 2015–2020. Results for SSP2-4.5, SSP3-7.0, and SSP5-8.5 show averages across the annual excess mortality computed based on climate-model-derived daily temperatures in 2095–2100. When we compute changes in excess mortality between 2020 and the end of the century (*SI Appendix*, Fig. S6), we compute changes within each climate model. We subtract average excess mortality computed based on the model-simulated temperatures in 2015–2020 from average excess mortality in 2095–2100. When presenting results for mortality on hot days, we focus on predicted excess mortality across days that exceed 20 ^°^C.

In robustness checks, we present results in which we allow the mortality response function of a county to evolve over time. We use observed 2005–2023 county characteristics $\mu_{c}^{T},\;\sigma_{c}^{T}$ and $RMSE_{c}$ to estimate a version of Eq. **1** with a coefficient vector from Eq. **2**. We use the same characteristics when projecting with static response functions. When allowing the response functions to evolve, we adjust the respective county characteristics to their 2095–2100 values. Adjusting $\mu_{c}^{T}$, for instance, allows the mortality-temperature-forecast-error response function of colder counties in 2015–2020 to become more similar to that of warmer counties in 2015–2020 as the average county temperature increases. To compute 2095–2100 county characteristics, we first compute the percentage change between 2015–2020 and 2095–2100 that the respective climate and forecasting scenario implies for each characteristic. We then apply this percentage change to the observed 2005–2023 characteristic. To avoid unreasonable extrapolations based on the linear functional form that we use for $\beta_{c}$, we restrict the evolving mortality response functions from going far out of sample: As in the estimation, we restrict future $\mu_{c}^{T}$, $\sigma_{c}^{T}$, and $RMSE_{c}$ to their respective 5th and 95th percentiles observed in-sample.

The change in county characteristics that we use to adjust the evolving mortality response functions is illustrated in *SI Appendix*, Fig. S7. It shows county characteristics at the 2005–2023 baseline in red and according to SSP3-7.0 and the expected trend forecast accuracy in 2095–2100 in blue. Panel (*A*) shows that changes in average temperature $\left(\mu_{c}^{T}\right)$ and daily temperature variability $\left(\sigma_{c}^{T}\right)$ are relatively modest and mostly in-sample. As shown on the vertical axis in panel (*B*), forecast accuracy in 2095–2100 goes substantially out of sample. We prevent such far extrapolations by restricting characteristics to lie within the 5th and 95th percentiles indicated by the black boxes in *SI Appendix*, Fig. S7. Even such restricted changes in RMSE lead to substantial uncertainty in results but similar point estimates, as shown in the last column of Fig. 5.

#### Deriving distributions of future forecast errors.

In our expert elicitation, we surveyed professional weather forecasters about the accuracy of temperature forecasts in 2005 and 2020. We also asked for their expectations regarding how accuracy will evolve by 2050 and 2100. For future years, respondents provided estimates of median accuracy, along with best- and worst-case scenarios, corresponding to the 1st and 99th percentiles of future accuracy. Using these responses, we construct probability distributions for forecast accuracy in 2050 and 2100.

Of the 46 experts who participated in our survey, 11 responded to the quantitative section. We found little evidence that these experts held systematically different opinions on key topics (Fig. 3) or that they were concentrated in a specific region.

Forecasters could express their assessments using one of three forecast accuracy metrics: Mean Square Error (MSE), Root Mean Square Error (RMSE), or Mean Absolute Error (MAE). As illustrated in *SI Appendix*, Fig. S11, there is a strong linear relationship between observed annual RMSE and MAE of forecast errors at the weather forecasting office level from 2005 to 2023. We leverage this relationship to convert MAE-based responses into their RMSE-equivalent values.

To analyze expected changes, we compute the 1st, 50th, and 99th percentiles of RMSE change for each expert by normalizing their 2050 and 2100 accuracy estimates with their reported accuracy for 2020. The raw responses are shown in *SI Appendix*, Fig. S12. To aggregate uncertainty across experts, we assume that each expert’s expected changes follow a skew lognormal distribution $F_{i}^{t}$, which is well suited for modeling percentage changes as it is strictly positive and accommodates asymmetric expert-reported percentiles. For each expert, we calibrate the mean, SD, and skewness of $F_{i}^{t}$ to align their reported percentiles with the theoretical percentiles of this distribution.

To obtain an overall distribution of expected RMSE changes by 2050 and 2100, we construct a mixture distribution: $F^{t}=\sum_{i}w_{i}F_{i}^{t}.$ In our baseline specification, we assign equal weights $w_{i}=1/N$ to each expert due to a lack of prior information favoring specific experts. We then derive the final RMSE distributions for 2050 and 2100 by scaling the observed U.S.-wide RMSE from 2020 by the corresponding distribution $F^{2050}$ or $F^{2100}$. These distributions, along with different future forecast accuracy scenarios, are displayed in Fig. 3.

In an alternative specification, we assess how well each expert’s reported RMSE change between 2005 and 2020 aligns with observed data. We adjust expert weights accordingly, increasing weights for those whose estimates are closer to observed values and decreasing them for those with greater discrepancies. The weighting follows an exponential scheme:

[7] $$\begin{array}{c} w_{i}=\frac{\exp(-\lambda {\left(y_{i}-y_{i,obs}\right)}^{2})}{\sum_{i}\exp\left(-\lambda {\left(y_{i}-y_{i,obs}\right)}^{2}\right)}, \end{array}$$

where $y_{i}$ is the change reported by expert i, and $y_{i,obs}$ is the observed change. With λ=20, experts closest to the observed 2005–2020 change are up-weighted by approximately 34%, whereas the expert with the poorest estimate receives 90% less weight compared to the equal-weight baseline. *SI Appendix*, Fig. S13 shows that the resulting future accuracy distributions remain relatively robust to this reweighting.

#### Analysis of open-ended expert responses.

Respondents were asked two open-ended questions about forecast quality. The questions asked about temperature forecasts: “Briefly: By 2050, what could make these forecasts more accurate?” and “By 2050, what could make these forecasts less accurate?” Respondents were also asked about communicating forecasts: “What do you believe are the biggest challenges for your organization in communicating weather forecasts to the public and public officials?”

To summarize the responses to the forecast quality questions, we counted the fraction of responses that contained words in six categories. The categories were determined based on manually reviewing responses and classifying common terms. Those categories and the terms they contain are as follows:

- *Observations & Data:* “observation,” “remote sensing,” “stations,” “sounders,” and “satellite”
- *Computing:* matches all terms starting with “comput”
- *Modeling:* “bias correction,” “ensemble,” “resolution,” and “parameter”
- *AI/ML:* “AI,” “ML,” spelled out versions of both, “QC,” and “oversight”
- *Funding:* “fund,” “resource,” and “budget”
- *Nothing:* “nothing” and “can’t think of anything”

Results showing the fraction of responses containing these terms are shown in *SI Appendix*, Fig. S14.

We conducted a semantic similarity analysis using the text package version 1.5 for R version 4.4.2. After cleaning each set of answers to remove nonwords, we calculated the semantic similarity using cosine distance for all answers across respondents within each question. This provided pairwise similarity measures for all respondents. The difference in average similarity across the forecast quality questions was tested using a two-sided t test.

## Supplementary Material

Appendix 01 (PDF)

## Data Availability

Anonymized CSV data and survey instruments have been deposited in OSF (53).
