# Supplementary material for: Weather forecasts become more important for reducing mortality as the climate warms
Source: Proc Natl Acad Sci U S A. 2026 Apr 13;123(16):e2523372123. doi: 10.1073/pnas.2523372123 (PMC13099585; doi:10.1073/pnas.2523372123)
Supplement: Supplementary file 1 — Appendix 01 (PDF) [file pnas.2523372123.sapp.pdf]

Supporting information for
Improving Weather Forecasts Saves Even More Lives Under Climate Change

Jeffrey G. Shrader, Stephan Thies, Laura Bakkensen, Manuel Linsenmeier, Derek Lemoine

Jeffrey Shrader Email: jgs2103@columbia.edu

This PDF file includes:

Figures S1 to S16

Additional Figures and Tables

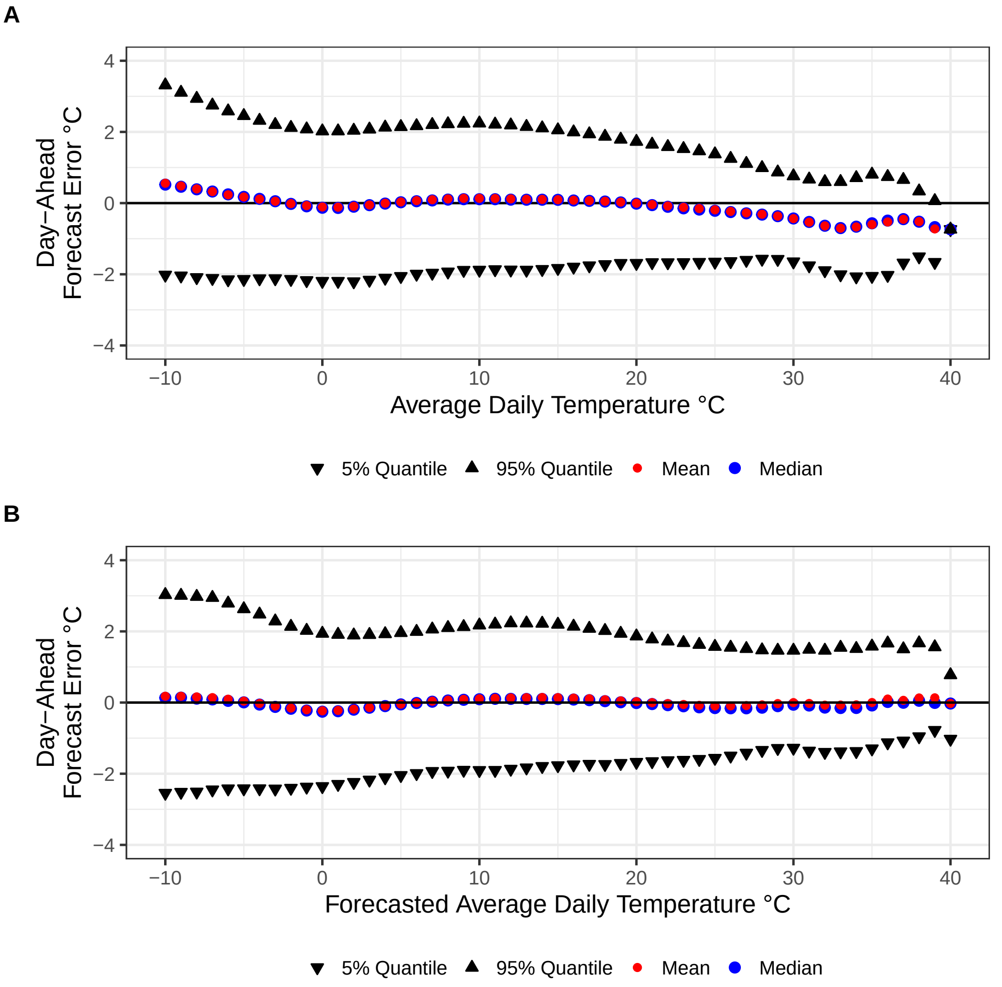

Figure S1: Forecast errors are relatively unbiased conditional on forecasted temperatures but biased conditional on realized hot and cold temperatures. The figure shows the distribution of day-ahead temperature forecast errors conditional on realized temperature (panel A) and forecasted temperatures (panel B). While forecast errors are unbiased conditional on forecasts (ex-ante), they are biased conditional on very hot and very cold temperatures (ex-post).

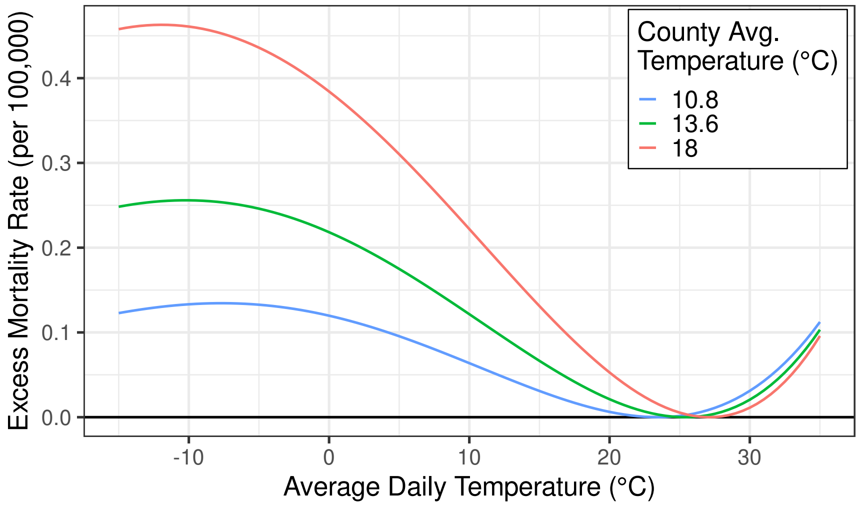

(a) Response Function Heterogeneity

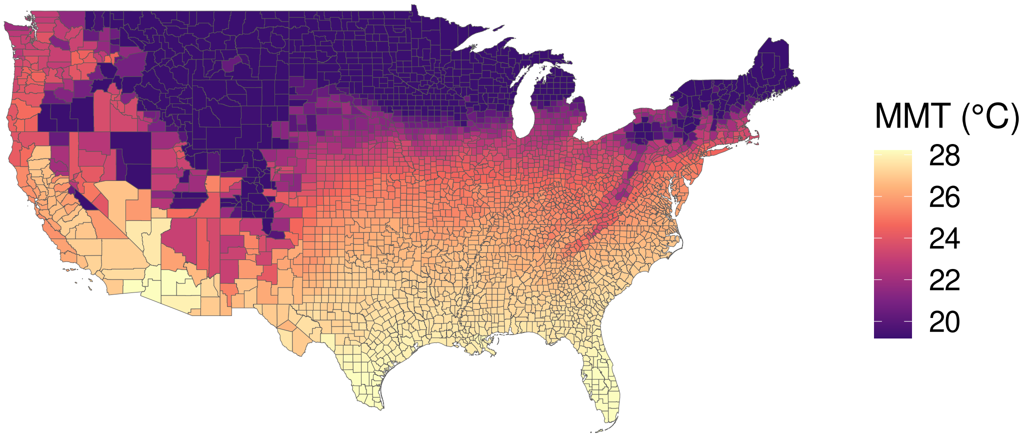

(b) Estimated Minimum Mortality Temperature by County

Figure S2: Heterogeneity in the mortality-temperature response function. The figure shows heterogeneity in the estimated temperature-mortality response function by county average temperature in panel (a) and resulting differences in the estimated minimum mortality temperatures in panel (b). The results show that higher average temperature in a county is associated with higher minimum mortality temperature, a higher mortality rate due to cold temperatures, and a lower mortality rate due to hot temperatures. See Figure 5 for results from models that allow this form of state dependence.

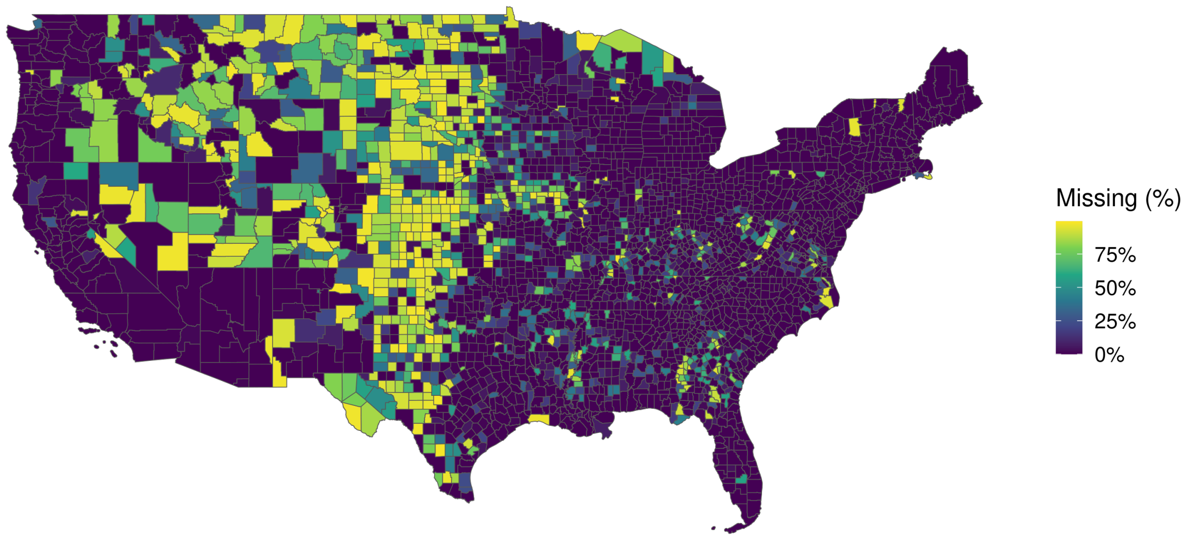

Figure S3: Percentage of suppressed values in CDC Wonder data. The map shows the percentage of suppressed and missing monthly deaths in the CDC wonder database for the period between 2004 and 2022. See Figure S15 for estimates based on the data only from counties with completely unsuppressed mortality records.

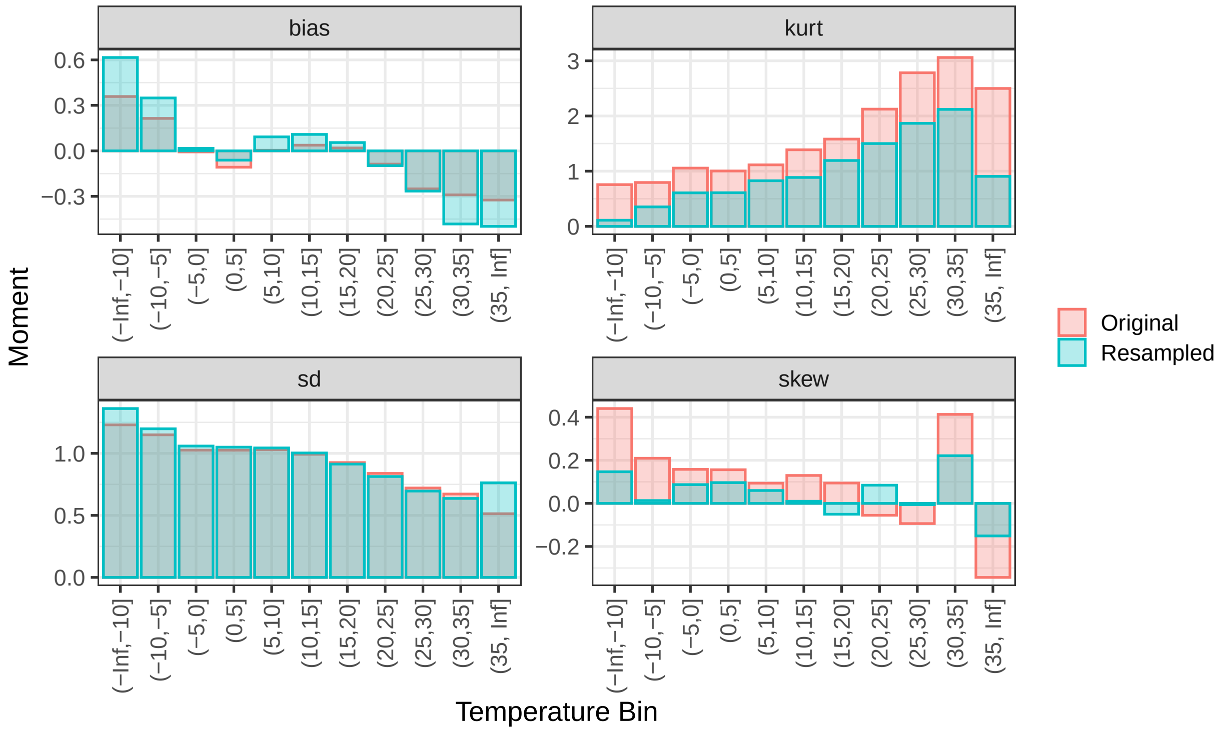

Figure S4: Resampled and original forecast error moments conditional on temperature. The figure shows observed population-weighted moments of forecast errors (red) as well as moments of resampled hypothetical forecast errors (blue) conditional on realized temperature in the observed 2015–2020 weather data that is used as the no climate change baseline.

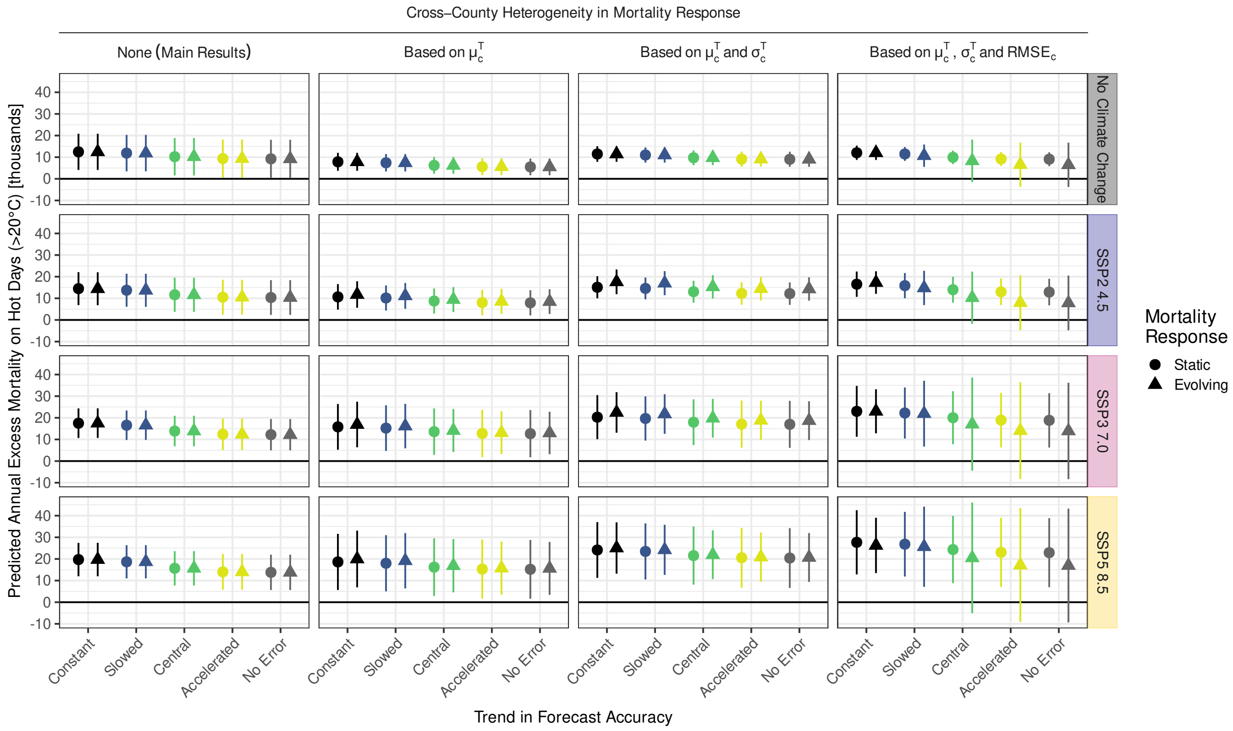

Figure S5: Annual excess mortality on hot days in 2100 across model specifications, forecasting, and climate change scenarios. The figure displays projections of the annual excess mortality on days exceeding 20°C average temperature under different scenarios for 2100 forecast accuracy, different model specifications, and climate change scenarios. Results in the no climate change scenario (row one) show annual averages based on observed 2015–2020 temperatures. Results in the remaining scenarios (rows two to four) show averages based on model-simulated 2095–2100 temperatures. Results in column one stem from a homogeneous mortality response function. Results in column two allow for cross-county heterogeneity in the mortality response based on average county temperature μ_c^T. Results in column three also allow for heterogeneity based on daily temperature variability σ_c^T. Results in column four also allow for heterogeneity along the average forecast accuracy in a county, measured as the RMSE of forecast errors. Circles show results for static response functions, and triangles show results for response functions that can evolve by the end of century based on changing county characteristics (see Methods for details).

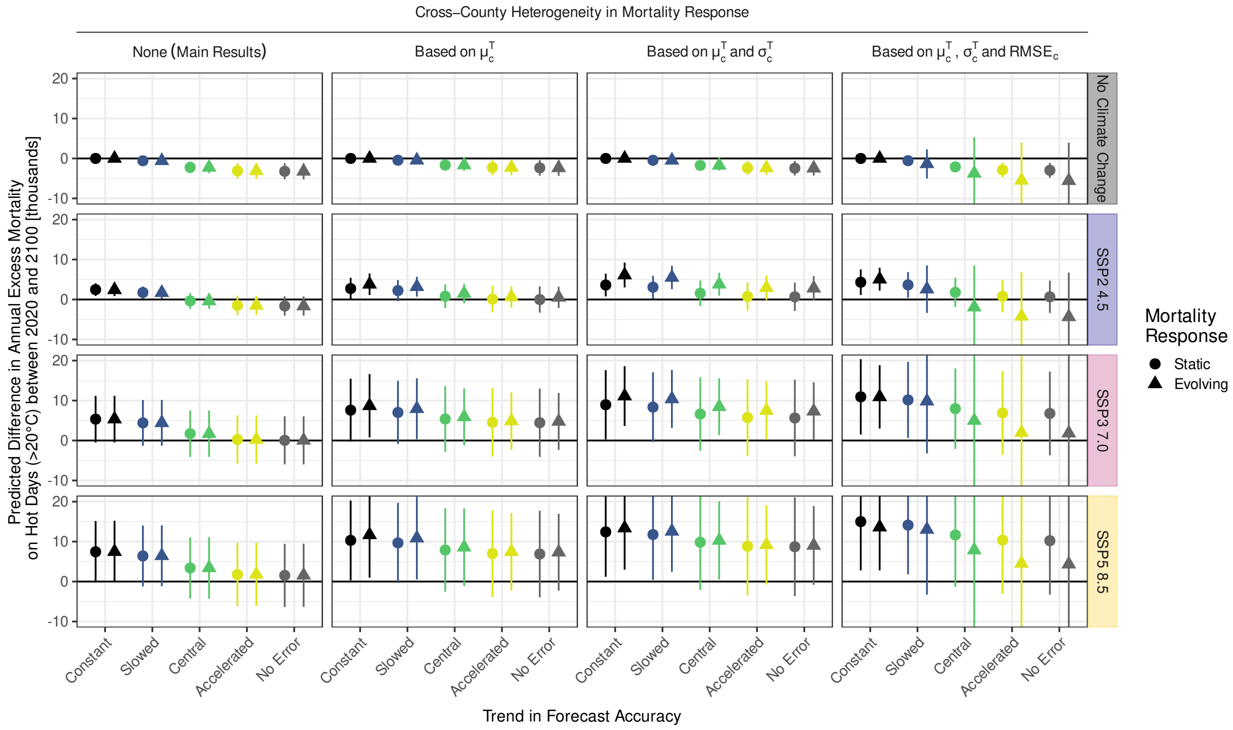

Figure S6: Projections of changes in annual excess mortality on hot days across models. The figure displays projections for changes in annual excess mortality on days exceeding 20°C average temperature between 2020 and 2100 under different model specifications and climate change scenarios. Negative numbers indicate lives saved while positive numbers imply an increase in mortality relative to the 2020 baseline. Results in column one stem from a homogeneous mortality response function. Results in column two allow for cross-county heterogeneity in the mortality response based on average county temperature μ_c^T. Results in column three also allow for heterogeneity based on daily temperature variability σ_c^T. Results in column four also allow for heterogeneity along the average forecast accuracy in a county, measured as the $RMSE_c$ of forecast errors. Circles show results for static response functions, and triangles show results for response functions that can evolve by the end of century based on changing county characteristics (see Methods for details).

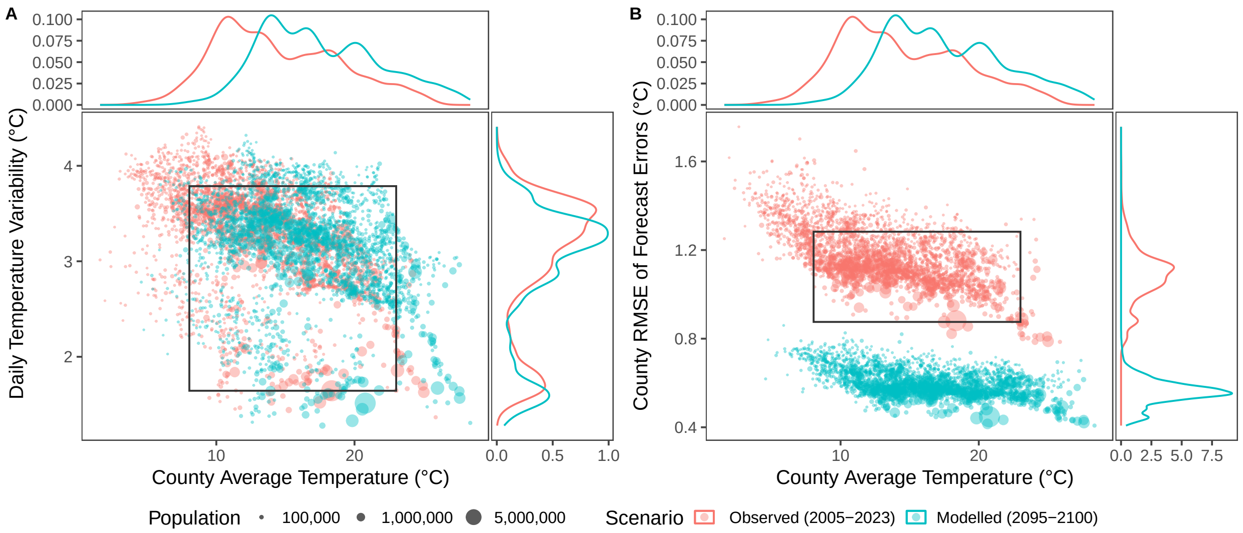

Figure S7: Changes in county characteristics according to SSP3-7.0 and expected improvements in forecast accuracy. The figure shows the distribution of the three county characteristics (average temperature μ_c^T, daily temperature variability σ_c^T, and forecast $RMSE_c$) in the observed 2005–2023 baseline data (red) and in 2095–2100 according to SSP3-7.0 and the *central* scenario for improvements in temperature forecast accuracy (blue). The black boxes show 5th-95th population weighted percentiles of each variable at baseline. When we allow mortality response functions to evolve by the end of century, we Winsorize county characteristics to stay within the boxes, preventing extrapolations far out of sample.

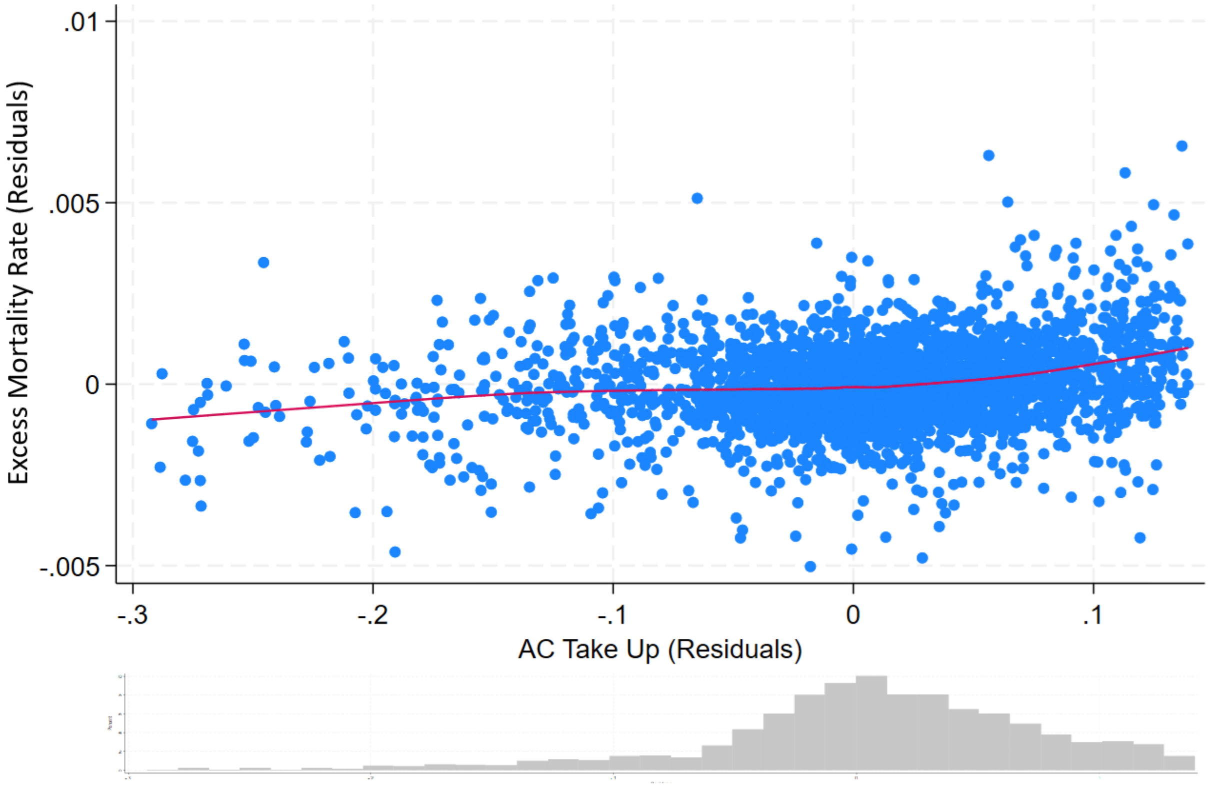

Figure S8: Air conditioning and excess mortality (hot days, >20°C) are positively correlated. The figure plots the relationship between residuals of air conditioner take-up (x-axis, in year 2017) and the residuals of the excess mortality rate from forecast errors (y-axis, estimated county average across data sample), after controlling each for county-specific average temperature (μ_c^T), temperature variability (σ_c^T), and forecast accuracy ($RMSE_c$). Each blue dot represents a U.S. county. To depict the main relationship, we include counties with residualized air conditioning rates within the 2.5th and 97.5th percentiles and omit the four lowest excess mortality residual counties. The red line displays the locally weighted scatterplot smoothing (lowess) relationship. The positive slope indicates a complementary association between the two variables. Below the x-axis is the histogram (gray bars) of the air conditioning take-up residuals.

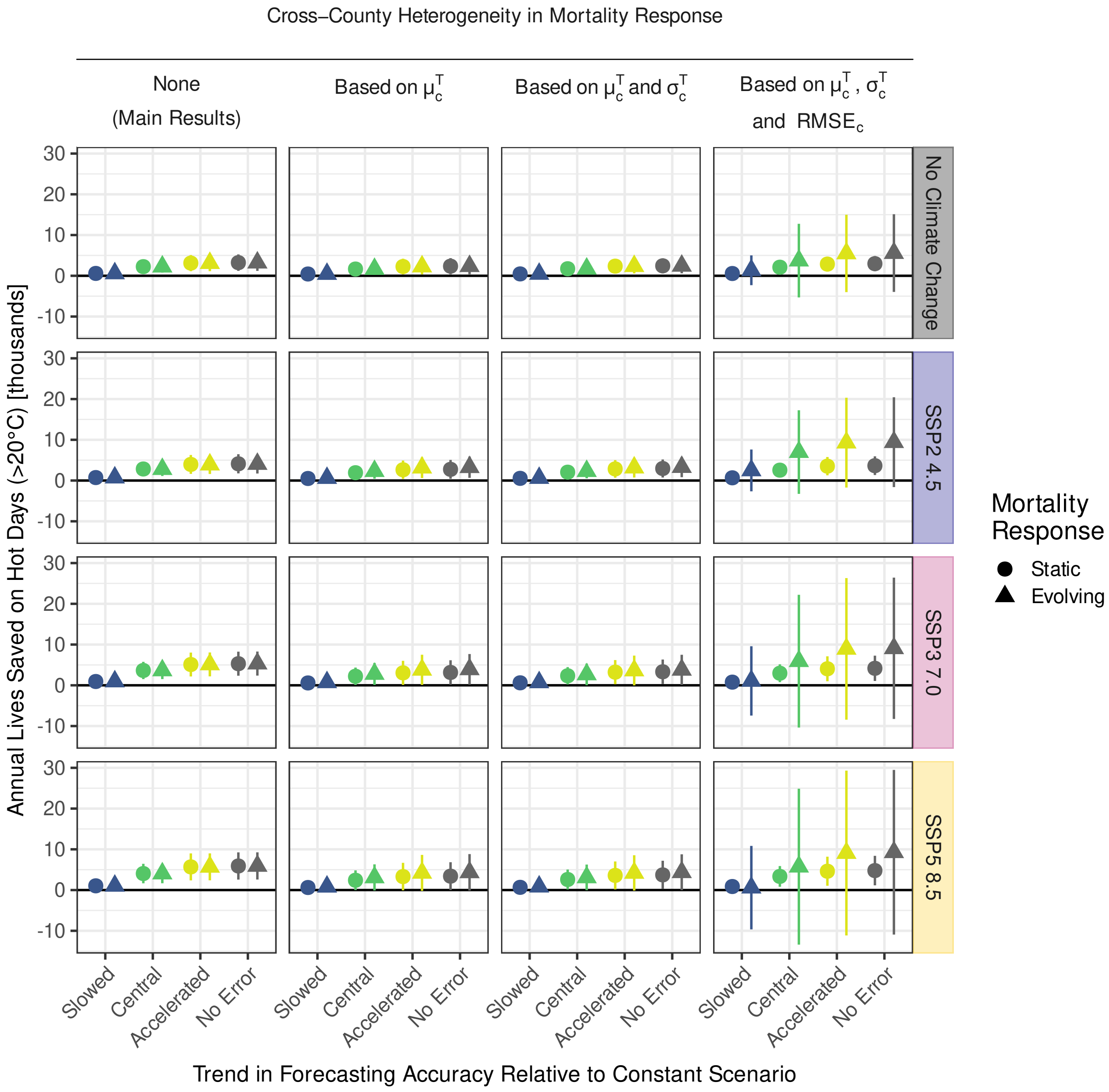

Figure S9: Replication of Figure 5 showing full confidence intervals. Compare with Figure 5 in the main text.

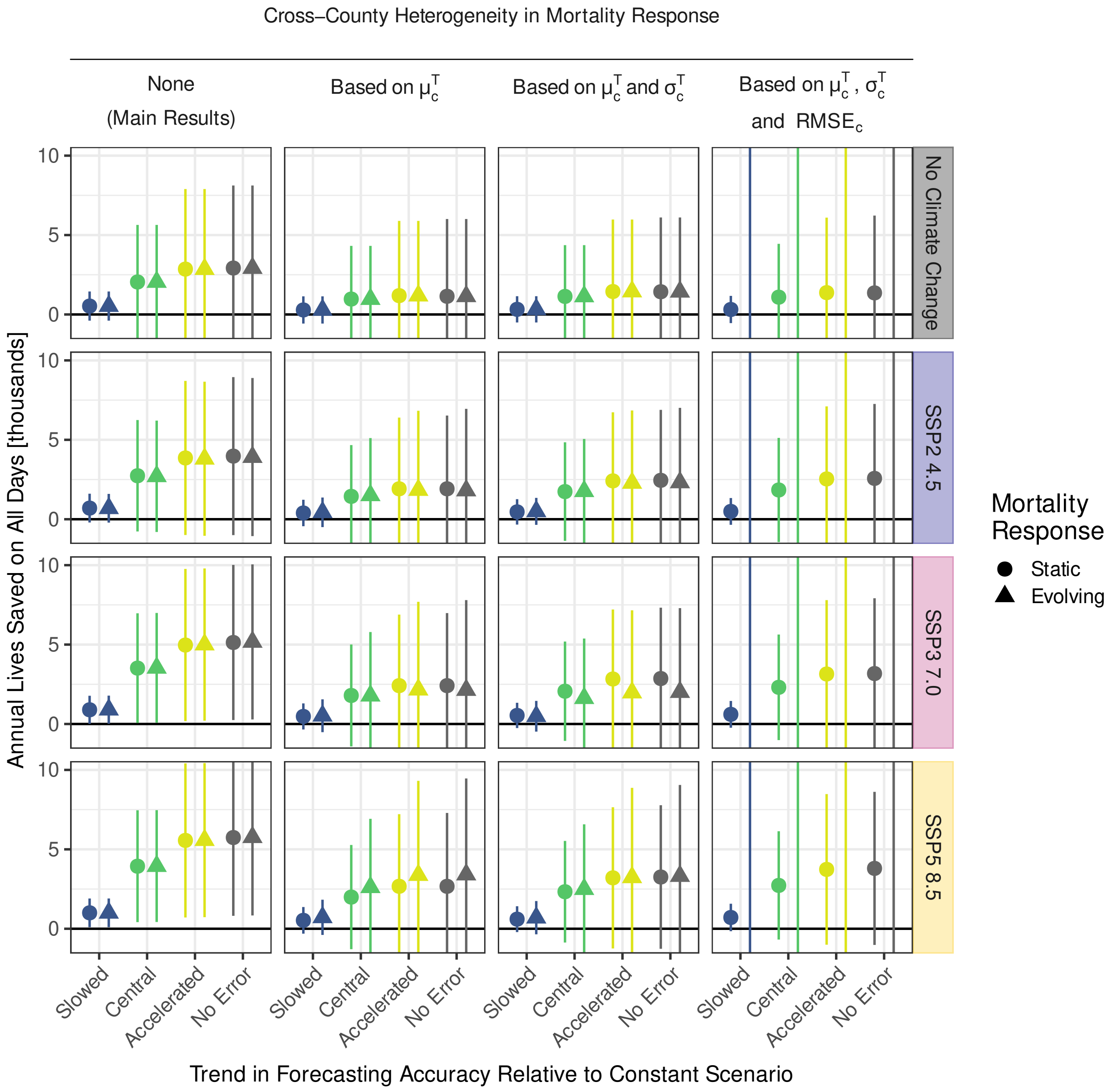

Figure S10: Version of Figure 5 showing results for mortality on all days. Compare with Figure 5 in the main text. This figure shows effects on all days.

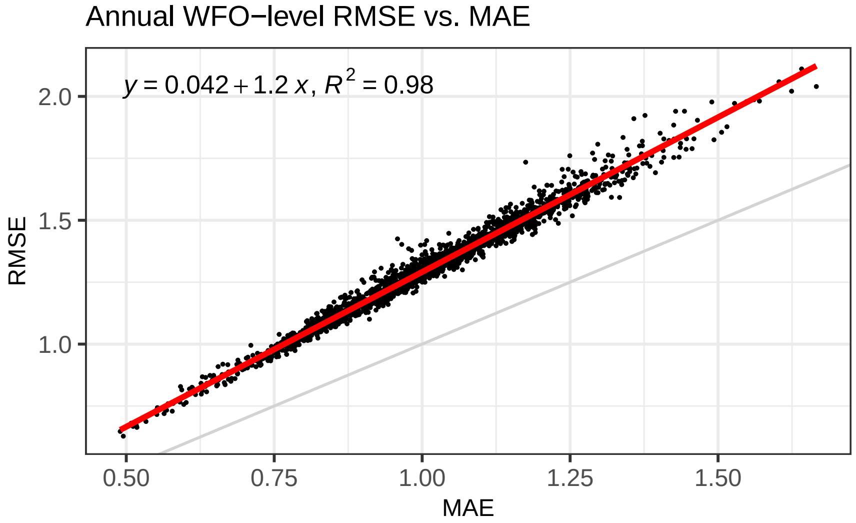

Figure S11: Root Mean Square Error (RMSE) and Mean Absolute Error (MAE) of temperature forecasts are linearly related. The scatterplot shows the empirical relationship between annual RMSE and MAE of one-day-ahead 2-meter temperature forecasts across weather forecasting offices (WFOs) in the contiguous U.S. between 2005 and 2023.

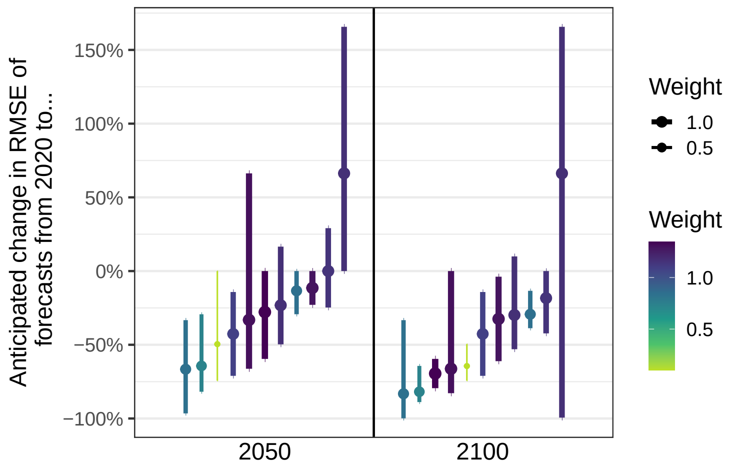

Figure S12: Expert expectations about changes in forecast error RMSE between 2020 and 2050 or 2100. Points in each figure show individual answers about the expected median change in RMSE between 2020 and 2050 or 2100. Vertical lines show the expert's 1st and 99th percentile predictions of future accuracy. Colors and line widths show to what degree answers are up- or down-weighted when we use quality weights.

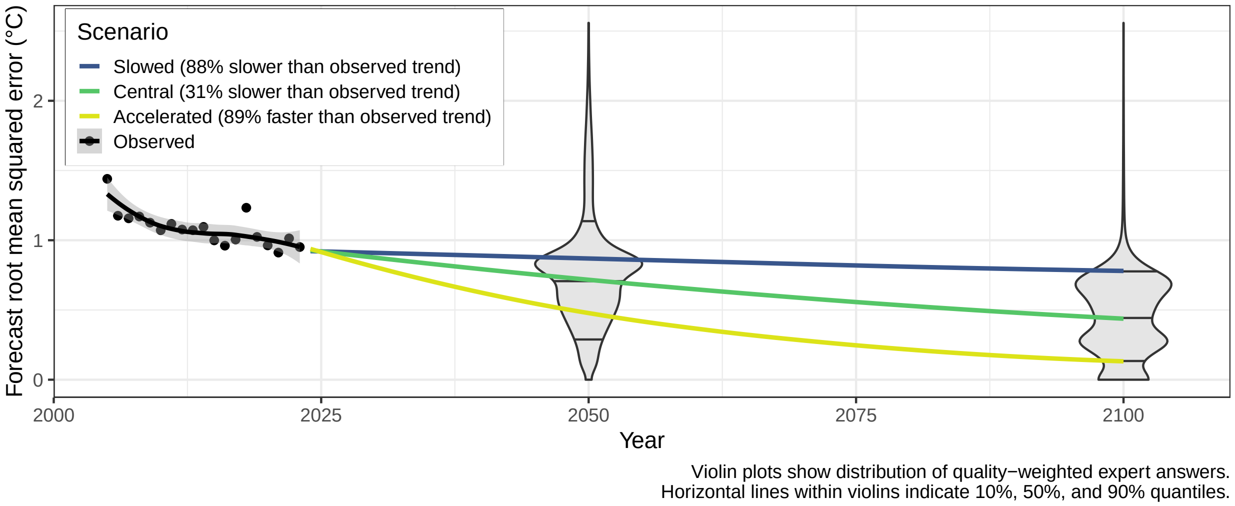

Figure S13: Forecast accuracy scenarios in comparison to quality-weighted expert distributions. Compare to Figure 3 A in the main text.

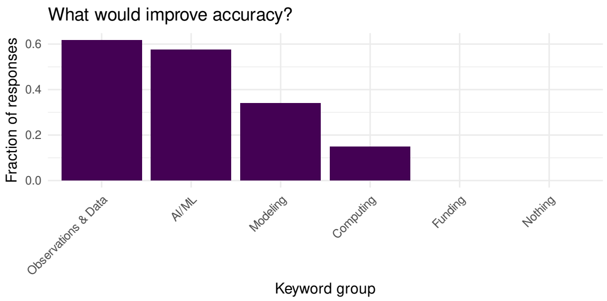

(a) What would improve forecasts

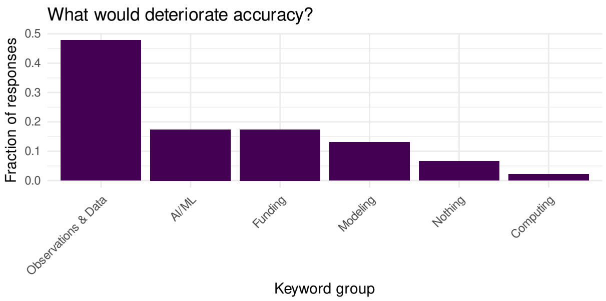

(b) What would degrade forecasts

Figure S14: Summary of responses to question about what would affect forecasts by 2050. The figures show summary information about a free response question posed to the full set of experts. The question asked experts what they thought could lead to forecast accuracy improvements (panel a) and deterioration (panel b) by 2050. The bars show the fraction of responses that contain terms in each of the groups listed on the x-axis. “Observations & Data” includes “observation,” “remote sensing,” “stations,” “sounders,” and “satellite.” “Computing” matches all terms starting with “comput.” “Modeling” includes “bias correction,” “ensemble,” “resolution,” and “parameter.” “AI/ML” includes “AI,” “ML,” spelled out versions of both, “QC,” and “oversight.” “Funding” includes “fund,” “resource,” and “budget.” “Nothing” includes “nothing” and “can’t think of anything.”

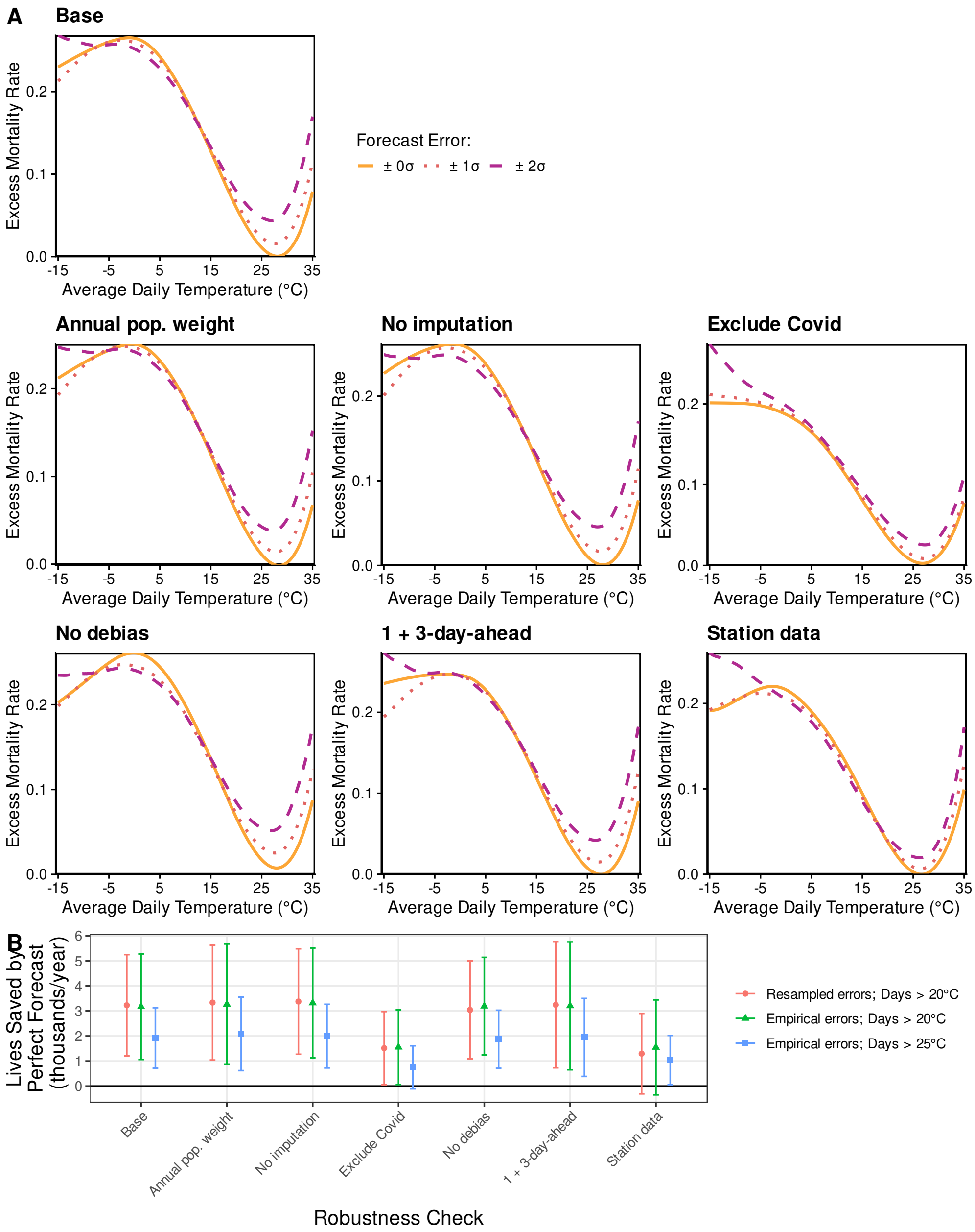

Figure S15: Robustness checks. The figure shows robustness checks and sensitivity analysis for the baseline results. Panel A shows versions of Figure 2 B for the baseline model plus models where: the beginning-of-sample population weights are replaced by annual population weights, all counties with imputed mortality are dropped, years after 2019 are dropped, forecast errors are not debiased, both 1- and 3-day-ahead forecasts are simultaneously included (and both are set to 0, 1, or 2 s.d. error for each line in the figure), and GHCN station data are used to calculate forecast errors. In all cases, we find that forecast errors increase mortality on hot days. Panel B shows estimates of the lives saved from reducing forecast errors to zero in sample. The red circles show versions of the rightmost estimate in the top-left panel of Figure 4 D (i.e., lives saved from zero-error forecasts on days above 20°C in the absence of climate change). Green triangles show the same when we use observed forecast errors rather than resampled errors. Blue squares again use observed forecast errors but calculate over days with average temperature above 25°C. Whiskers are 95% confidence intervals.

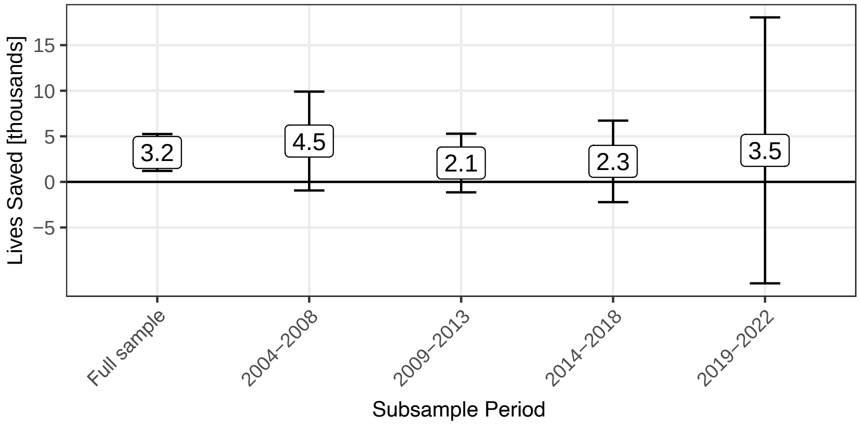

Figure S16: Comparing in-sample baseline estimate and estimates from subsample periods. The figure shows estimates of the lives saved from completely eliminating forecast errors in sample (i.e., without climate change) for the baseline model (leftmost point) and for models estimated on subsample periods of the data (date ranges given on the x-axis). All controls are the same in each model. Whiskers are 95% confidence intervals.

Table S1: Attrition Balance: Omnibus F-test

	Completion = 1
Public vs private advances	0.064
	(0.075)
Effect of AI	-0.265
	(0.117)
Effect of public funding	0.119
	(0.075)
Effect of staffing	0.025
	(0.058)
Effect of climate change	-0.089
	(0.090)
N	42
F-stat	1.904
p-value	0.109

This table shows balance tests comparing the completers (dependent variable equals 1) to the respondents who started but did not complete the survey (dependent variable equals 0). The right-hand-side variables ask about the effect on future forecast accuracy of public versus private sector forecasters, AI, public funding of national weather services, staffing levels at national weather services, and climate change, all on a Likert scale converted to numeric responses. Estimated coefficients are displayed, with heteroskedasticity robust standard at the participant level shown in parentheses beneath each coefficient. The omnibus F-statistic tests balance between the two groups; p-value based on randomization inference following recommendations from [29].
